# Predicting Variabilities in Cardiac Gene Expression with a Boolean Network Incorporating Uncertainty

**DOI:** 10.1371/journal.pone.0131832

**Published:** 2015-07-24

**Authors:** Melanie Grieb, Andre Burkovski, J. Eric Sträng, Johann M. Kraus, Alexander Groß, Günther Palm, Michael Kühl, Hans A. Kestler

**Affiliations:** 1 Leibniz Institute for Age Research, Fritz-Lipmann Institute, Jena, Germany; 2 Core Unit Medical Systems Biology, Ulm University, Ulm, Germany; 3 Neural Information Processing, Ulm University, Ulm, Germany; 4 Institute for Biochemistry and Molecular Biology, Ulm University, Ulm, Germany; 5 International Graduate School of Molecular Medicine, Ulm University, Ulm, Germany; Technische Universität Dresden, Medical Faculty, GERMANY

## Abstract

Gene interactions in cells can be represented by gene regulatory networks. A Boolean network models gene interactions according to rules where gene expression is represented by binary values (*on / off* or {1, 0}). In reality, however, the gene’s state can have multiple values due to biological properties. Furthermore, the noisy nature of the experimental design results in uncertainty about a state of the gene. Here we present a new Boolean network paradigm to allow intermediate values on the interval [0, 1]. As in the Boolean network, fixed points or attractors of such a model correspond to biological phenotypes or states. We use our new extension of the Boolean network paradigm to model gene expression in first and second heart field lineages which are cardiac progenitor cell populations involved in early vertebrate heart development. By this we are able to predict additional biological phenotypes that the Boolean model alone is not able to identify without utilizing additional biological knowledge. The additional phenotypes predicted by the model were confirmed by published biological experiments. Furthermore, the new method predicts gene expression propensities for modelled but yet to be analyzed genes.

## Introduction

Specialization of cells during development and differentiation is driven by transcription or growth factors. These are interconnected in gene regulatory networks. The temporary regulated interaction of these factors are finally resulting in terminally differentiated, specialized cells which are characterized by the expression of a certain set of genes. Thus, development and function of a certain cell type is largely reflected by the expression of selected genes in a cell. Gene regulatory networks describe the interactions between those genes in the cell [1–3]. During embryonic development, these gene regulatory networks evolve over time towards a stable state, finally reflecting the terminally differentiated cell [1], i.e., biological phenotypes.

A gene regulatory network can be visualized as a static map that describes the interaction of these genes and reflects the activation or inactivation of genes by other factors in the network. Such a gene regulatory network can be implemented as a Boolean network if one assumes that a gene can be either active or inactive in a cell and thus can be represented by a Boolean value (*on* / *off* or {1,0}). Interaction between genes can then be mathematically modeled by Boolean functions. A set of such logical rules or functions, more exactly one Boolean function per considered gene defines a Boolean network (BN) [4, 5]. Given some initial expression pattern, a BN computes the evolution of gene expression in discrete time steps. Of particular importance are states which are invariant or lead to periodic sequences of expression patterns, so called attractors. For finite sized BNs any initial state will converge to one of these attractors in finite time [6] In a Boolean network representing a gene regulatory network, these attractors are the equivalent to the stable state of gene expression reflecting the differentiated biological phenotype of the cell.

BNs are useful as a first approach when it comes to model complex networks with many genes and their interactions [7]. Often the BN is modeled from known regulatory interactions that are manually derived from qualitative wet-lab experiments [8] or computationally determined with BN reconstruction methods [9, 10]. Additionally, simulated Boolean states of genes from the simulation allow an intuitive interpretation of the results. Recently, BN models have been used to capture the essence of gene regulation in several biological processes such as the mammalian cell cycle [11], the guard cell abscisic acid signaling [12], or the oxidative stress response pathway [13].

Modelling of gene regulatory networks and their simulation, however, is hampered by different drawbacks. In practice, for example, absolute data for gene expression activities are measured indirectly, e.g., by quantifying the relative amounts of the corresponding transcripts. These measurements are inherently noisy. Furthermore, some notion of activity/inactivity has to be inferred in order to infer the state of the gene. To this effect binarization schemes are used in order to differentiate between active and inactive genes in time series data [14]. Here, one also has to consider that effective thresholds are gene dependent [8]. Finally, one has to take into account that gene expression can vary between different cells of an apparently homogeneous population of cells as previously shown for the common cardiac progenitor cell population that gives rise to the heart [15].

Here, we implement a novel extension of the Boolean network paradigm and illustrate the procedure on a Heart Field Development model. We also illustrate the utility of the method on the Mammalian Cell Cycle [11] (see Section G in S1 Supplementary Information File). Our primary focus is the evolutionary conserved core cardiac regulatory network that drives early cardiac development and that predicts the dynamic behaviour of gene expression during early development [16]. In this process a common cardiac progenitor cell population splits into two populations of cells called first (FHF) and second heart field (SHF), that are characterized by the selective expression of typical transcription factors such as Isl1, Tbx1, Tbx5, and Nkx2.5. Cells of the FHF develop to the primary heart tube and later to the left ventricle and the atria, whereas cells of the SHF mainly develop into the right ventricle and the outflow tract [17]. Activation of these genes during cardiac development is regulated among others through growth factors of the Wnt family [18]. The BN model of this gene regulatory network correctly predicts the general pattern of gene expression in general, work in Xenopus [19] and in mouse ES cells [15] albeit suggests that during cardiac development on a single cell level a much more complex variability in gene expression exists.

In an attempt to include the variability of single cell gene expression in an otherwise homogenous cell population, our goal was to describe gene expression levels with multiple values, extending the binary values of a BN. Previously, transformations of BNs have also been considered by others [20, 21]. An overview over these approaches can be found in [22, 23]. These transform the Boolean rules into a system of ordinary differential equations which describe the dynamics of gene concentrations. As opposed to the analysis of behaviour and evolution of concentration levels we use an approach to model continuous intervals of gene expression values in order to potentially find new fixed points using only the original BN. In our model, we call continuous values attributed to genes “propensities” of gene expression and do not need additional parameters that are otherwise required for modelling concentrations. The corresponding interactions are derived from the Boolean functions of the BN. The operations AND, OR, and NOT are replaced by their arithmetic counterparts based on fuzzy logic product sum rules [24]. This extends the BN into a discrete-time, non-linear, dynamical model that is represented by a system of difference equations, namely, a BN extension (BNE). Naturally, fixed points of this new model also represent possible expression patterns or phenotypes of cells. Interestingly, this novel method is able to predict variabilities in gene expression during cardiac development and cell cycle (see Figs M and N in S1 Supplementary Information File). For cardiac development the extension predicts additional phenotypes that are in agreement with published results and novel gene expression pattern for yet to be analyzed genes.

## Methods

Since we intertwine biological and mathematical terms we shortly present an overview of the important terminology that we use in the following:

- **Boolean fixed point:** A fixed point of the BN. It takes values $B^{n}\in {\left\{0,1\right\}}^{n}$ with *n* being the number of variables in the BN.
- **BNE fixed point:** A fixed point of the BNE. It takes values $I^{n}\in {\left[0,1\right]}^{n}$.
- **biological phenotype:** A phenotype that describes a binarized gene expression of measurements. It takes values in $B^{n}$, i.e., in form of binary gene expression.
- **hypothetical phenotype:** A pattern of gene expression. Hypothetical phenotypes are used in order to map the extension fixed points to their nearest neighbouring pattern. In our particular case we consider binary gene expression patterns with values in $B^{n}$ (a set of 2^*n*^ binary patterns).

**Fig 1:**
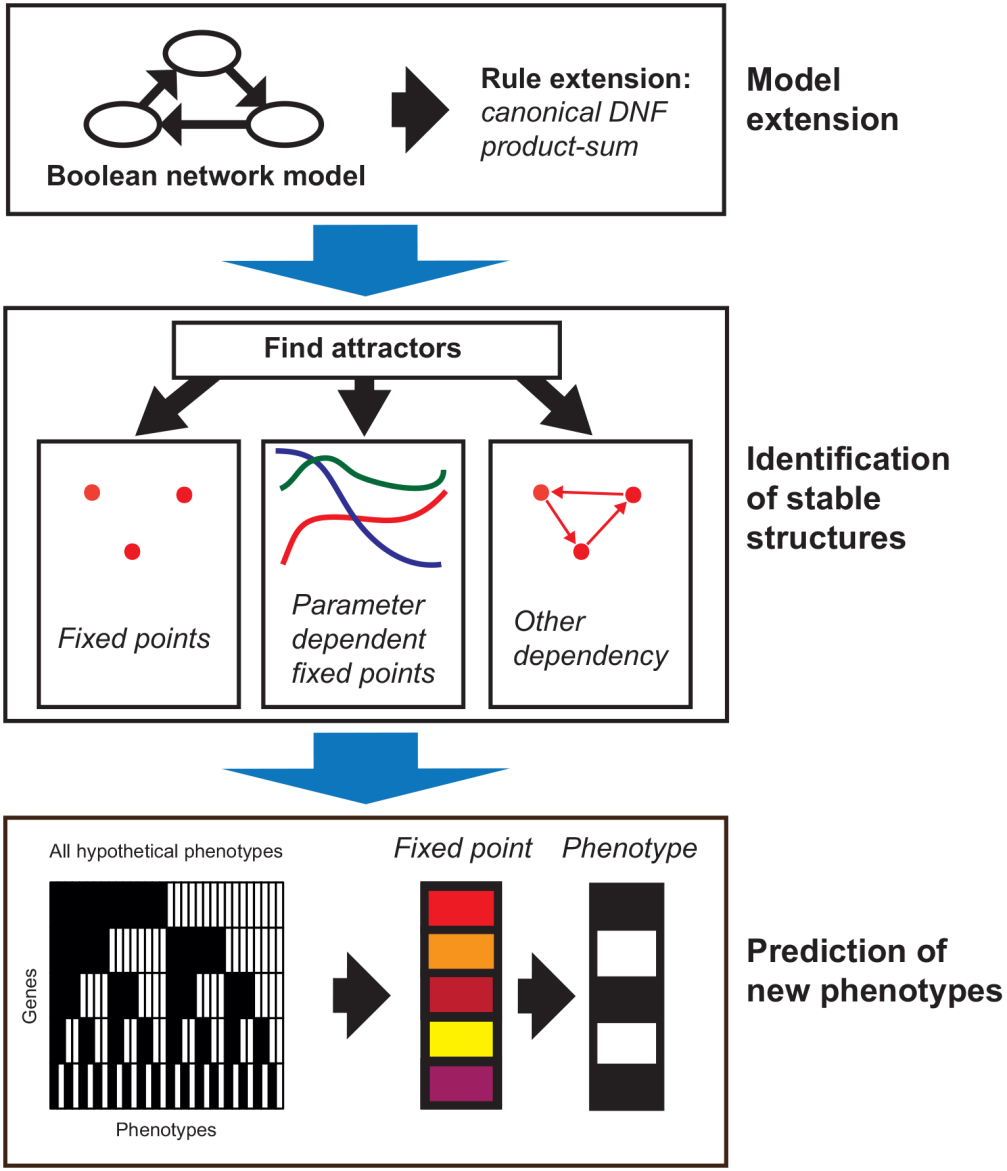
Phenotype analysis using the Boolean Network Extension (BNE). The application of the BNE to a given Boolean network (BN) can be divided into three basic steps: Model extension (top), identification of stable structures (middle) and mapping to phenotypes (bottom). In the model extension step (top) the rules of the BN are transformed to the rules of the BNE by converting the rules of the BN to canonical disjunctive normal form (DNF) and then to product-sum fuzzy logic (DNF product-sum extension, details see section Extension of Boolean networks). In the “identification of stable structures”-step (middle) the extended BNE is simulated for a large number of random inputs. The resulting approximated attractors can either be fixed points approximated by point clouds, fixed points depending on one or multiple parameters or different dependencies. Finally, new phenotypes are identified step (bottom) by mapping the fixed points to their nearest hypothetical phenotype.

The extension and analysis of the BN is conducted in several steps drafted in Fig 1 and are described in detail below. Given a BN, using the canonical Disjunctive Normal Form (DNF), each term is then transformed into a sum of products. The extended model is then simulated to find BNE fixed points of the model. These fixed points can be interpreted by a mapping the fixed points to the nearest hypothetical phenotypes. The meaning of the nearest hypothetical phenotype must then be further interpreted in biological context.

### Boolean Networks (BN)

BN were pioneered by Kauffman in 1969 [25, 26] to model gene interactions in a cell. BNs are based on the assumption that a cell regulates its function in a time-dependent manner by switching genes on (active) or off (inactive). The regulatory mechanism is described by logical rules where the state of a gene is defined by logic rules based on the previous gene states.

Here, we denote by 𝔹 = {0, 1} the set of Boolean values in binary representation. Formally, a BN is defined by *n* Boolean variables $x=\left(x_{1},\ldots ,x_{n}\right)\in B^{n}$ and a vector of *n* Boolean transition functions *F* = (*f*_1_, …, *f*_*n*_), which describe the interaction between variables.

A transition function *f*_*i*_ is a map *f*_*i*_:𝔹^*n*^ → 𝔹. In that way that the discrete time evolution generated by a BN is defined by sequential application of the vector valued function *F* = (*f*_1_, …, *f*_*n*_) on an initial state **x** at time 0. A state transition is thus formally described by the map

$$\begin{array}{c} x\;(t+1)=F(x(t)),t\in N_{0}, \end{array}$$ (1)

given the state **x**(*t*) at time *t*. 𝔹^*n*^ is finite and discrete and there are 2^*n*^ unique states. Each is a priori allowed as BN initial value. It follows that evolution of an initial state will converge to a cyclic sequence of states in finite time. Such sequences are called attractors. Attractors are usually categorized in

- fixed points (attractors of period 1) and
- periodic sequences (attractors of period *T* > 1).

In the following synchronous BN will be considered. In synchronous BN, each Boolean function is executed once at each time step.

In a Boolean network any variable may interact with any subset of variables in the network. Additionally, variables with constant values may be considered in order to parametrize external inputs, e.g., influences of exogenous factors. Discrete time delays may also be modeled by introducing a chain of “dummy” variables which consecutively take the value of the chain’s first variable value. Such a chain of *n* additional “dummy” variables result in a time delay of *n* steps of the considered variable.

### Boolean Network Extension (BNE)

By 𝕀 we denote the interval [0, 1]. The new model extends the state space from 𝔹^*n*^ to 𝕀^*n*^. We do so by adapting the logical rules of the original BN. In general one has to find a mapping from the Boolean operators AND (∧), OR (∨), and NOT (¬) to continuous operators (functions) on 𝕀^*n*^. For the sake of consistency, these operators should render the same results as their corresponding Boolean analogue when restricting the values to {0, 1}.

A common approach is to treat the Boolean transition functions as fuzzy logic functions [24, 27]. In the fuzzy logic literature, there are mainly two approaches. The first approach is the min–max fuzzy logic. The second approach, product–sum fuzzy logic, replaces the Boolean algebraic operators on 𝔹^*n*^ (∨, ∧,¬) with their arithmetic counterparts on 𝕀^*n*^, namely, (+, ×) and negation by (1 − *x*). Constant values of the BN are translated to constant functions of the BNE from 𝕀. Contrarily to the min–max fuzzy logic, the product–sum fuzzy logic are differentiable functions. Additionally, product–sum allows a smooth evolution and interaction between the variables which correspond the idea that interactions in cells depend on the concentration levels of products. In contrary, in min–max variables that are not minimal or maximal do not influence the resulting value. In the following, we will only consider the product–sum fuzzy logic.

Formally, the extension of a Boolean function

$$\begin{array}{c} f_{i}:\;{𝔹}^{n}\to 𝔹 \end{array}$$ (2)

$$\begin{array}{ccc} & & a\mapsto b_{i} \end{array}$$ (3)

is the function

$$\begin{array}{c} {\hat{f}}_{i}:\;{𝕀}^{n}\to 𝕀 \end{array}$$ (4)

$$\begin{array}{ccc} & & \hat{a}\mapsto {\hat{b}}_{i}, \end{array}$$ (5)

i.e., we consider the extensions of the Boolean domains and functions given by the operation $\hat{•}$. The product–sum fuzzy logic results in the following properties

$$\begin{array}{c} \hat{\neg x_{i}}=1-{\hat{x}}_{i}, \end{array}$$ (6)

$$\begin{array}{c} \hat{x_{i}\wedge x_{j}}={\hat{x}}_{i}\cdot {\hat{x}}_{j}, \end{array}$$ (7)

$$\begin{array}{c} \hat{x_{i}\vee x_{j}}={\hat{x}}_{i}+{\hat{x}}_{j}-{\hat{x}}_{i}\cdot {\hat{x}}_{j}. \end{array}$$ (8)

Eq 8 can be derived from Eqs 6 and 7 using DeMorgan’s law. The Boolean formulae are transformed into a unique representation using the canonical disjunctive normal form (DNF) which can be directly derived from the truth table. Then, given a Boolean formula in DNF, we directly extend it into the continuous version by applying the product-sum rules. The resulting formula have values in I since DNF encodes a truth table for every possible term with *n* variables [28]. It is easily verified that the BN and its extension coincide for variable values in $B^{n}$ when applying the DNF/product–sum extension (DNFPS).

**Example**: sequence of transformations for the Boolean function *f*(*a*, *b*, *c*) = (*a*∨*b*)∧*c*,

$$\begin{array}{lllll} \text{Boolean}\;\text{function} & \to & \text{DNF} & \to & \text{DNFPS}\\ \left(a\vee b\right)\wedge c & \to & \left(\neg a\wedge b\wedge c\right)\vee \left(a\wedge \neg b\wedge c\right)\vee \left(a\wedge b\wedge c\right) & \to & \left(\hat{a}+\hat{b}-\hat{a}\cdot \hat{b}\right)\cdot \hat{c} \end{array}$$

### Extension fixed points

As in the case of BN, its extension will have fixed points. Fixed points of the model are meant to represent biological states since both are stable and time-invariant. Firstly, we defined the extension procedure in such a way that a BN and its extension are consistent when restricting to values in $B^{n}$. Hence, the BN fixed points are fixed points of its extension as well. Secondly, an extension may have non–Boolean fixed points.

**Fig 2:**
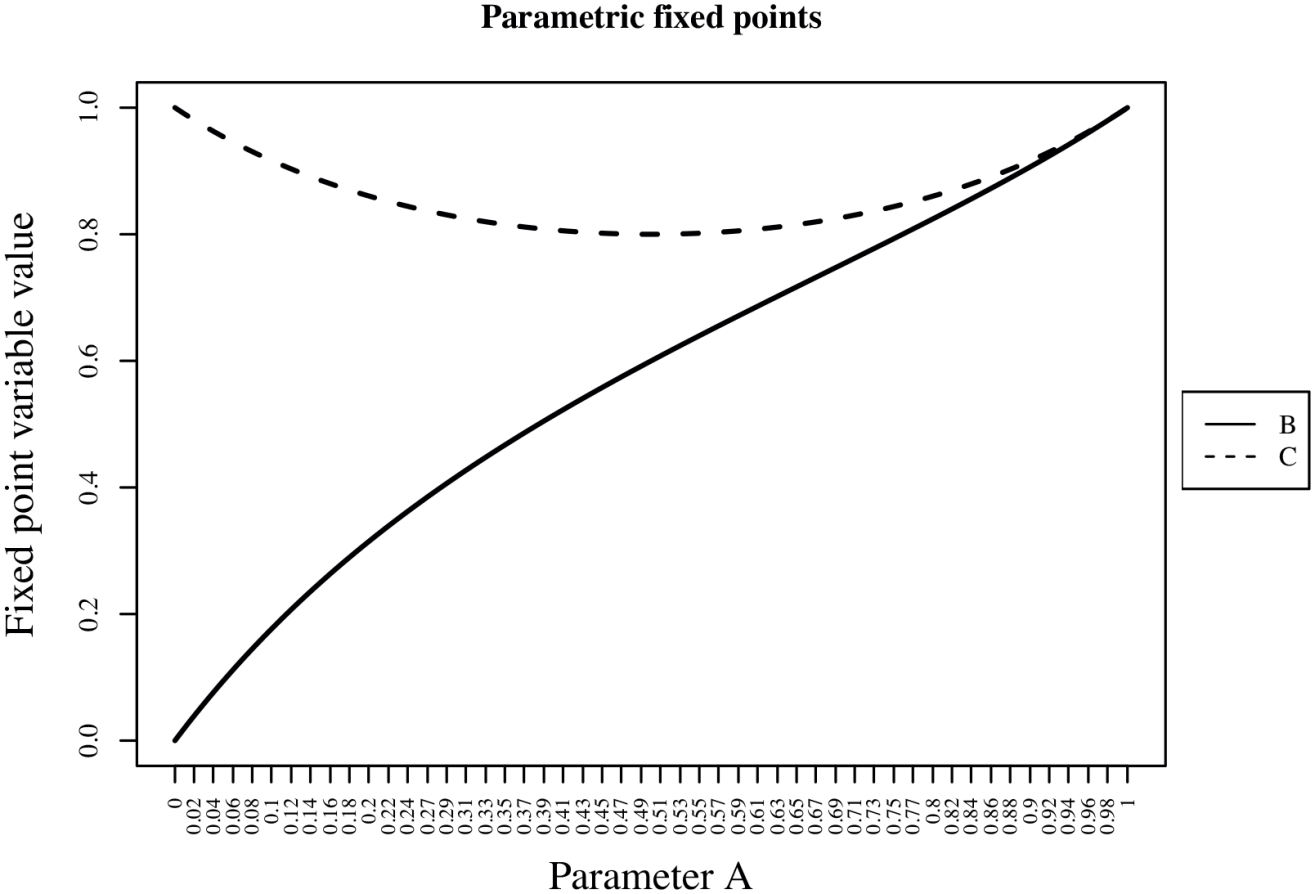
Parametric fixed points. An example of parametric dependency of fixed points is shown. Table 1 shows the BNE.

One crucial point of the extension is the fact that the variables of the system, i.e., variables which are considered as constant input, may now take values in I. If these parameters are uncertain, unknown, or the behavior of the system is to be examined, one would have to investigate the variations of the fixed points as the input variables are changed. Compared to the BN case, two new aspects need to be taken into account. It is a priori not possible to predict the number of fixed points of a BNE nor is it possible to conduct an exhaustive search. Additionally, the iteration of a BNE can only be (practically) carried out numerically. Thus the values of the fixed points given by iteration are not exact. The same would be true if the fixed points were found by numerically solving $\hat{f}\left(\hat{\text{x}}\right)=\hat{\text{x}}$. Any attempt to systematically find the set of extension fixed points of an extension would hence result in a numerical approximation of fixed points gained by sweeping the initial values of the respective search through $I^{n}$. Amongst those found, several may correspond to a single actual fixed point. A proper identification of the true fixed point is therefore necessary. They can, e.g., be grouped into fixed point prototypes by using k-means. The fixed points of the BNE would typically describe surfaces over the investigated parameter space (see Fig 2).

**Table 1:** Boolean Network Extension. For a given parameter $\hat{A}$, the BNE converges towards a single fixed point. The gene expression values $\hat{B}$ and $\hat{C}$ of the fixed point depend on this parameter. The x-axis corresponds to the parameter $\hat{A}$ and the y-axis shows the value of the fixed point for the variables $\hat{B}$ and $\hat{C}$, respectively. E.g., the fixed point for $\hat{A}=0.5$ is ($\hat{B}=0.6,\hat{C}=0.8$).

Boolean Network		Boolean Network Extension	
*A*	constant parameter	$\hat{A}$	constant parameter
*B*(*t*+1)	*A*∨¬*C*(*t*)	$\hat{B}(t+1)$	$\hat{A}+(1-\hat{A})\cdot (1-\hat{C}(t))$
*C*(*t*+1)	¬*A*∨*B*(*t*)	$\hat{C}(t+1)$	$\left(1-\hat{A})+\hat{A}\cdot \hat{B}(t\right)$

### Numerical considerations

BN were simulated with the help of the *BoolNet* package [29]. Fixed points were investigated in two manners

- iteration with initial data taken from uniform distributions over 𝕀^*n*^. The criteria for termination were met if the last *k* = 2 function evaluations of the iteration were below a threshold *ɛ* = 10^−8^ ($|{\hat{F}}_{t}(\hat{s})-{\hat{F}}_{t-1}(\hat{s})|<\varepsilon$) with $\hat{s}\in {𝕀}^{n}$ being the propensity vector. The maximal number of iterations for the heart field model and cell cycle model was 100 since a typical convergence was achieved after 20–30 function evaluations.
- numerically solving
  $${𝕀}^{n}∍\hat{s}?:\hat{F}(\hat{s})=\hat{s}$$ (9)
  using the *nleqslv* R-package [30]. More specifically, we used the Broyden secant method [31] which is a heuristic for the Newton method. The global strategy uses the *dbldog* argument which is a the trust region method using a double dogleg method [32].

Both methods yielded similar results up to solver tolerance. Graphics were generated with R [33].

### Association of BNE fixed points with putative biological phenotypes

Whether considering prototypes or actual fixed points of the extension, the actual predictive value of the model is given by its capacity to describe and predict biological phenotypes. As mentioned above, we expect our model to reflect the properties of a Boolean state under perturbation. It would hence be expected that only stable fixed points may be of interest biologically since stable states correspond to biological states in equilibrium. Also, in simulations, unstable fixed points are unlikely to be found numerically. However, known Boolean fixed points may turn out to be unstable under the extension. Thus the BNE is able to additionally characterize the stability of the Boolean fixed points. Conversely, new stable fixed points may be found by the BNE.

The values of the fixed points do require a scheme to determine expression levels of each specific gene to enable comparison with measured binary biological phenotypes. In practice this would mean that we would need a binarization scheme to extract the gene expression in terms of a set of a priori unknown critical values. For this reason we employ a more absolute scheme for the identification by mapping fixed points and hypothetical phenotypes which lie nearest to each other in $I^{n}$, in a subset of $S_{I}\subset I^{n}$, or a fuzzy description of hypothetical phenotypes in {0, 1}^*n*^. We call this identification nearest neighbor matching (NNM). In general, using a distance measure results in a specific ordering of the considered elements. Typically we use the Euclidean distance since it is widely known and has an intuitive interpretation. In order to assess the mapping approach we also applied it in context of the cell cycle network [11] (see Section G in S1 Supplementary Information File).

### Interpretation of the values of a BNE

The BNE uses fuzzy logic product-sum transformation of the Boolean rules to compute a value for each variable. The main property of the extension is that it *inherits the interaction patterns of the BN*. The defined operations corresponding to the Boolean operations are also *t*–norms and corresponding associated co-norms, which ensure that the formalism is a consistent fuzzy logic [34, 35]. Furthermore, the extension is conducted in such a way that the transition of the DNFPS to the Boolean limit is differentiable.

We wish to emphasize that the assumption we make is that the approximation also yields plausible results for larger perturbations over the entire $I^{n}$ and that the intermediate values will be in a monotonic relation to measurements of gene expression, i.e, the larger the value *x*_*i*_ the larger the expression of the corresponding gene product.

The assertions of the BNE values are in no way absolute but do reflect the corresponding expression of genes in a relational way, i.e., place the corresponding expressions on an ordinal scale. We can state, a gene has a “higher” or “lower” expression values when comparing two values. This however does not give any conclusive answer to whether a gene is expressed or not. The values only reveal an ordering of gene expressions. From the Boolean case we expect that 0 corresponds to certainly unexpressed and 1 to certainly expressed. An actual quantification for values in between 0 and 1 may hence only be carried with some a priori knowledge, i.e., by comparison with actual measurements.

As the BNE attributes numbers to genes, it might also be natural to interpret those as concentrations. An example would be to associate the outputs of the BNE as the gene transcript products over time. However, it is very unlikely that, what in effect is a system of kinetic reactions may be described without the addition of any kinetic parameters. Such attempts have been carried out in the past and do require additional parameters to describe the dynamics of the underlying chemical molecules [20, 21]. Since the values are neither concentrations nor probabilities we call them *propensities* for gene expression.

## Results

### Previous results for early cardiac development

Herrmann et al. [16] analyzed the gene regulatory network of early cardiac development by use of a BN and found a correspondence between biological measurements and mathematically simulated attractors. The model simulates the interaction between intracellular genes (Bmp2, canonical Wnt, Dkk1, Fgf8, Foxc1.2, GATAs, Mesp1, Isl1, Nkx2.5, Tbx1, Tbx5) and extracellular factors (exogenous Bmp2 and canonical Wnt) that form gradients (Table 2).

**Table 2:** Genes, proteins and model variables to BN model of cardiac development. The first column shows the variables used in the BN model. The second column, function, describes the type and location of the expressed protein or the purpose of the variable in the BN.

Variable (Gene/Protein)	Function
**Intracellular factors**	
*Bmp*2 (Bmp2)	Signaling factor
*canWnt* (canonical Wnt)	canonical Wnt signaling
*Dkk*1 (Dkk1)	Signaling factor
*Fgf*8 (Fgf8)	SHF transcription factor
*Foxc*1.2 (Foxc1, Foxc2)	SHF transcription factor
*GATAs* (GATA4, GATA5, GATA6)	transcription factor, cardiogenic mesoderm
*Isl*1 (Isl1)	SHF transcription factor
*Mesp*1 (Mesp1, Mesp2)	transcription factor, early cardiogenic mesoderm development
*Nkx*2.5 (Nkx2.5)	transcription factor, cardiogenic mesoderm
*Tbx*1 (Tbx1)	SHF transcription factor
*Tbx*5 (Tbx5)	FHF transcription factor
**Extracellular factors**	
*exogen*_*Bmp*2_*I*	Bmp2 derived from neighboring tissue
*exogen*_*Bmp*2_*II*	Time delay of Bmp2 derived from neighboring tissue
*exogen*_*CanWnt*_*I*	Canonical Wnt derived from neighboring tissue
*exogen*_*CanWnt*_*II*	Time delay of canonical Wnt derived from neighboring tissue

When considering all possible binary initial values, two fixed points were reached in 99% of the cases. The gene expression values corresponding to these two fixed points are similar to the gene expression in the FHF and SHF that was extracted from literature. In 49% of the initial values, the network converges to attractors corresponding to the FHF (49% of cases) and in 50% to the SHF. In the remaining 1% the simulation converged towards a fixed point with no activated cardiac genes. This was thought to correspond to a biological phenotype where no heart field is formed, like if canonical Wnt signaling is not activated during development. The BN fixed point that resembles the FHF is characterized by the expression of the FHF specific genes Bmp2, GATAs, Nkx2.5, and Tbx5 to be active. Accordingly, the fixed point for the SHF shows activation of the SHF genes, Isl1, Foxc1/2, Tbx1 and Fgf8. In the following we name the phenotypes predicted by the Boolean model *FHF*_*BOOL* and *SHF*_*BOOL*, respectively. We apply the BNE to this cardiac development model in order to investigate new phenotypes that are outside of the Boolean pradigm.

**Fig 3:**
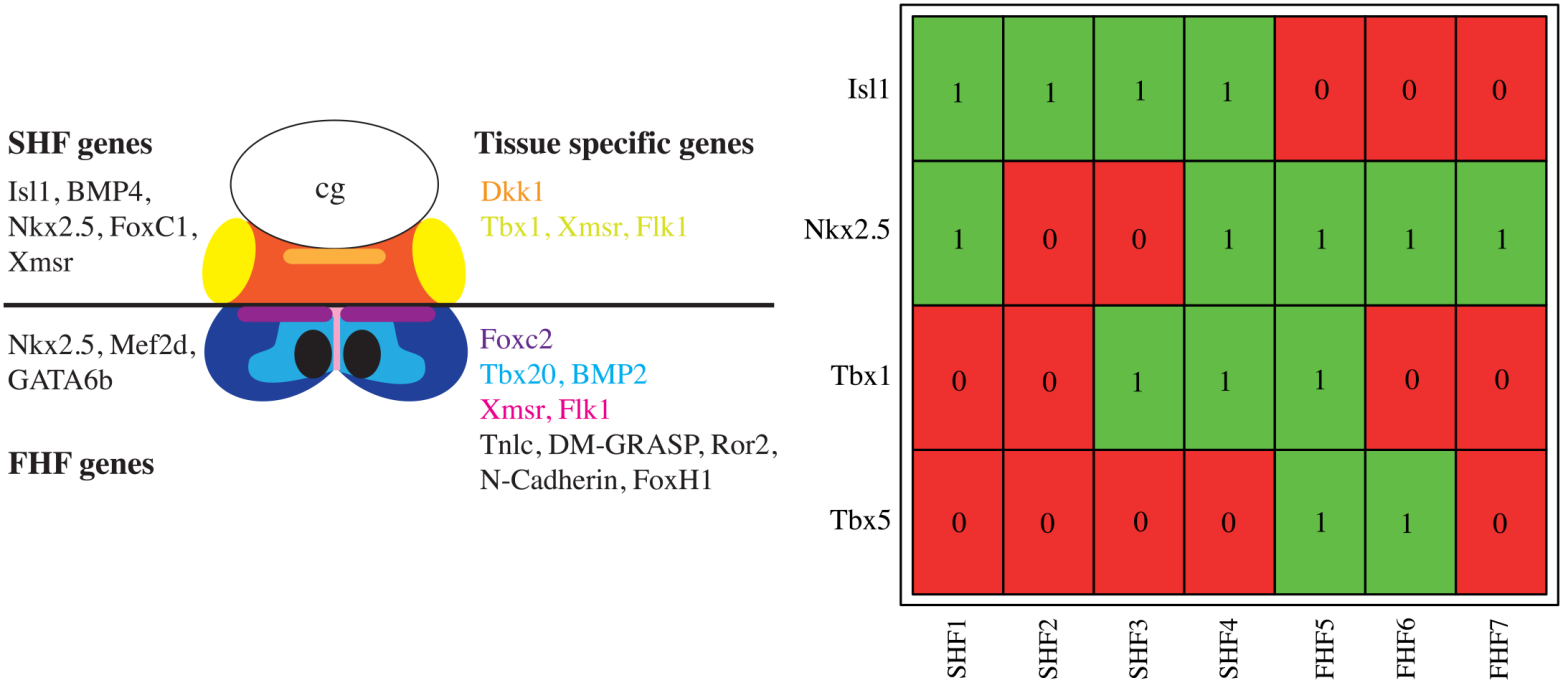
Schematic drawing of cardiac tissue in Xenopus laevis at stage 24—Expression of genes in heart fields (left) and RT–PCR analysis of selected genes (right). Panels adapted from Gessert and Kühl [19]. The left panel shows the genes expressed in different domains of the first heart field (FHF) and second heart field (SHF). The SHF is shown at the top and the FHF is shown at the bottom. Common genes expressed in all regions of the FHF and SHF, respectively, are shown on the left. Genes expressed in particular domains are shown on the right. Colors indicate different domains and corresponding expressed genes. The right figure shows the results of single cell RT–PCR analysis of gene expression for the four genes Nkx2.5, Isl1, Tbx1, and Tbx5. Values (0 and 1) and colors red/green represent inactive or active genes. The panel shows the gene expression of different single cell samples (numbered and named at the bottom). FHF and SHF are distinguished by the expression of the Isl1.

Biological phenotypes are described in terms of present or absent gene expression. In our case we wanted to compare the gene expression of the four genes Isl1, Nkx2.5, Tbx1, and Tbx5 for FHF and SHF differentiation to the single cell RT-PCR analysis of Gessert and Kühl [19]. The phenotypes previously identified are given in Fig 3. In the following we name these phenotypes as SHF1, SHF2, SHF3 and SHF4, as these are determined as second heart field phenotypes by the expression of the SHF marker gene Isl1, and the phenotypes representing the first heart field FHF5, FHF6, FHF7.

### Simulation results for the BNE

Given the continuous model of early cardiac development we computed fixed points for 10^4^ different initial values of the genes. The values for each gene were drawn from a uniform distribution *U*(0,1). We additionally performed simulations that were aimed at initial inactivation (value 0) of all genes with the *exogen*_*canWnt*_*I* as controlling parameter in order to examine its influence to the formation of the phenotypes. As the cardiac model is parametrized by the *exogen*_*canWnt*_*I* parameter, we ordered the identified fixed points accordingly. The linear increase of the propensity of the *exogen*_*canWnt*_*I* causes a continuous transition in propensity for the remaining genes (see Fig B in S1 Supplementary Information File).

### Prediction of additional phenotypes for FHF and SHF

The structure provided by the BN is sufficient enough to allow a prediction of previously unknown biological phenotypes through the extension.

In order to characterize the phenotypes in the BNE we compared all 11 genes that are effectively modeled by the BN (Bmp2, canonical Wnt, Dkk1, Fgf8, Foxc1.2, GATAs, Mesp1, Isl1, Nkx2.5, Tbx1, Tbx5). This allows prediction of gene expression propensity of genes for which no expression information is available. In order to evaluate our results we computed the distances between all hypothetical phenotypes and the continuous fixed points for each parameter value of *exogen*_*canWnt*_*I* in the simulation. The 11 genes give rise to 2^11^ = 2048 different qualitative expression patterns that are compared and the phenotype with the minimal distance to a fixed point is then considered to be the phenotype of this fixed point. In order to distinguish the hypothetical phenotypes we use the naming scheme “*PH-X*” where X is the decimal number encoding the binary expression pattern of the corresponding phenotype.

**Fig 4:**
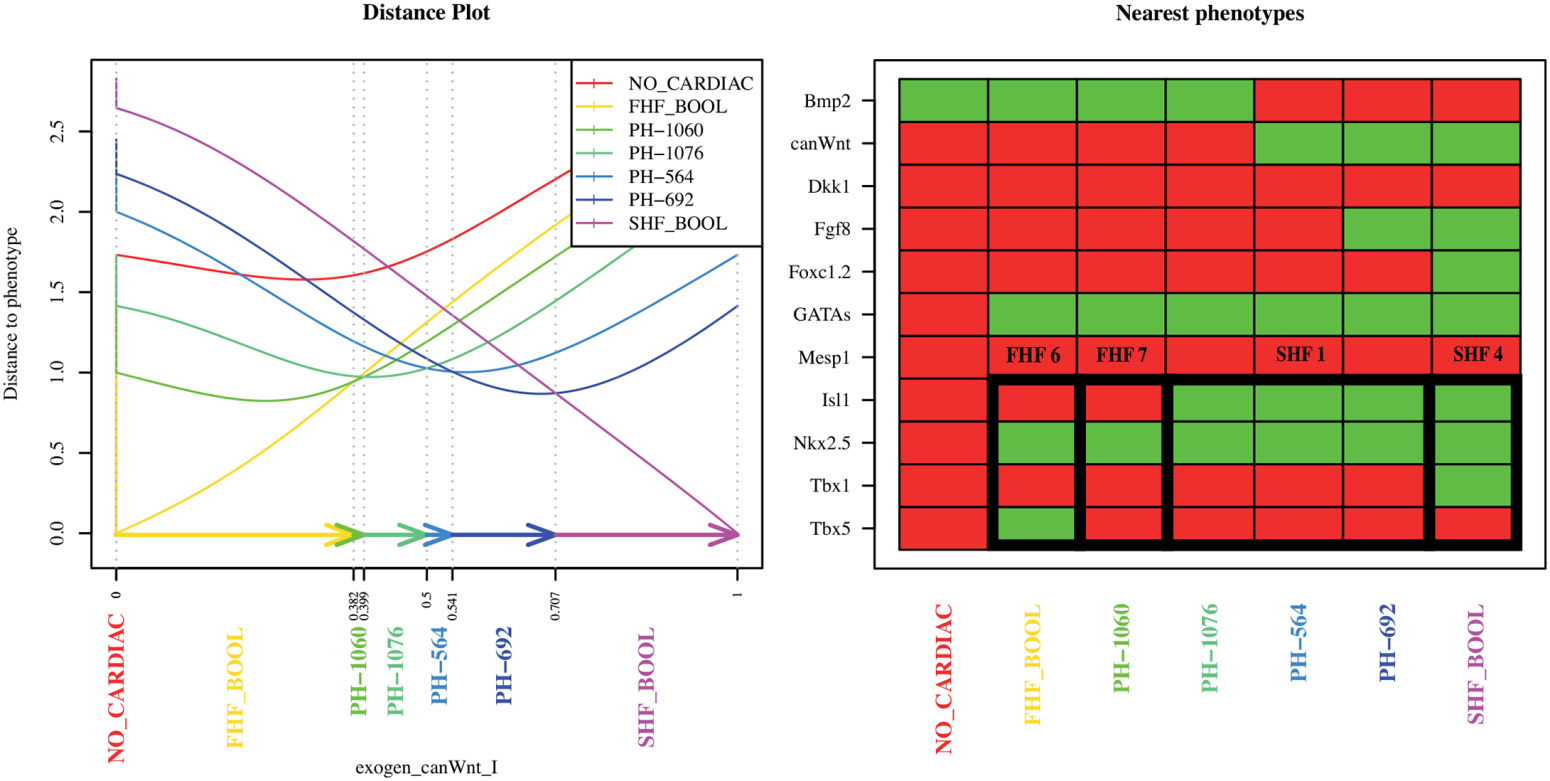
Phenotypes predicted by the BNE. The phenotype profile used for the mapping is based on the 11 genes present in both the Boolean model and the *Xenopus* analysis. The figure in the left panel shows the distance curves for the nearest phenotypes and fixed points. The x-axis denotes the values of the parameter *exogen*_*canWnt*_*I* and the phenotypes to which the fixed points were mapped. The y-axis shows the actual distance. The phenotypes are ordered by increasing *exogen*_*canWnt*_*I* expression propensity (right panel). Activated genes are shown in green and deactivated genes are shown in red. The framed box shows the gene expression propensity pattern for the four genes Isl1, Nkx2.5, Tbx1, and Tbx5 that corresponds to the the RT–PCR phenotypes of the FHF and SHF from the Fig 3. The *FHF*_*BOOL* and *SHF*_*BOOL* phenotypes correspond to the phenotypes found in the Boolean model [16]. The SHF1 phenotype is split in three sub-phenotypes *PH-1076*, *PH-564*, and *PH-692* that differ by the gene expression propensity of canWnt, Bmp2, and Fgf8. The expression of the Fgf8 gene was not reported in *Xenopus*. Its activation pattern is a prediction of the BNE.

Fig 4 shows the 7 nearest hypothetical phenotypes that are mapped to the computed fixed points of the BNE. For the genes Isl1, Nkx2.5, Tbx1, and Tbx5 we see the same expression propensity pattern that is referenced in the RT-PCR analysis. The phenotypes FHF6 and SHF4 also correspond to the fixed points of the BN model *FHF*_*BOOL* and *SHF*_*BOOL*, respectively. The *NO*_*CARDIAC* fixed point of the BNE also corresponds to the “no-cardiac” fixed point of the BN. For the remaining phenotypes BNE predicts expression pattern for the FHF7 via *PH-1060* and additionally three new phenotypes. These phenotypes, *PH-1076*, *PH-564*, and *PH-692*, correspond to the SHF1 based on the four genes measured by RT-PCR [19], however, the data shows that the SHF1 phenotype may have different gene expression propensities for the genes Bmp2, canonical Wnt, and Fgf8 (Fig 4).

### Stability of fixed points of the BN

The Boolean phenotypes predicted by the BN model correspond to the FHF6 and SHF4 phenotypes (Figs 3 and 4). In the BNE these phenotypes correspond to values close to 0 and 1 of the *exogen*_*canWnt*_*I* expression propensity. Consistently, our extension can predict the same phenotypes of the BN model and it shows that under the perturbation of *exogen*_*canWnt*_*I* parameter the results remain close to the phenotypes found by the BN model. The 1% attractor, which was assumed not to correspond to any of the heart fields, is also a fixed point of the BNE. In our simulation we additionally analyzed the BN fixed points for stability. In particular, the 1% attractor reported in the Boolean Network model. Any perturbations added to this fixed point resulted in the fixed point corresponding to FHF4 for the given value of *exogen*_*canWnt*_*I*. This fixed point is thus unstable in the BNE.

## Discussion

We extended the Boolean network model of early cardiac development and identified the biological phenotypes that were previously predicted by the Boolean model as well as additional biological phenotypes that represent a more detailed differentiation of the FHF and SHF in terms of gene expression propensities. These additional phenotypes were confirmed by experiments in *Xenopus laevis*[19].

There are several advantages of the proposed method. Essentially, it helps to characterize the gene expression propensity of phenotypes from the structure given by the BN alone. It corresponds to the BN model in case of binary inputs. The complexity of the extension can be seen as an intermediate representation between the BN and ODE models. It can cope with continuous values but does not need additional kinematic parameters that are required, e.g., in an ODE model for concentration levels. Considering the possibility theory approach [36], the BNE does not use fuzzy sets and thus is not representable as a possibility. In our case, the fuzzy logic approach is used to extend the Boolean function on the intervals [0, 1].

Different approaches exist that transform a BN into a system of ordinary differential equations. Mendoza and Xenarios [21] partition the genes into activating and inhibiting subsets. Each subset is postulated to activate or inactivate a considered gene in a sigmoid manner adding the corresponding terms to the ODE vector field. They do not directly transform the logical rules of the BN, but rather construct the rules according to the activator and inhibitor subsets. They identify fixed points in the system by perturbing the Boolean attractors. By focusing on activation and inhibition of the genes they limit the BN to only a subset of possible Boolean rules for state transition. Conversely, the approach of Wittmann et al. [20] directly transforms the rules of a BN into continuous functions. They include production and decay rates into the rules, thereby introducing additional parameters into the model. Transformation of the continuous input variables to continuous switch-like values is done with Hill functions [37–39]. Both approaches have in common that they require additional knowledge either from biological experiments or expert opinion to determine the values for the different parameters.

The state space of BN is discrete and finite. The attractors can be exactly determined by simulating the network in an exhaustive manner. In this respect, the BNE has the same limitation as an ODE model. In general, the complete exploration of the search space for identification of fixed points is infeasible and we cannot determine the number of fixed points in the system a priori. Fixed points of the model can only be found numerically by sweeping through the search space.

The BNE behaviour resembles that of the Boolean model for small perturbations in the Boolean input. When the Boolean input is perturbed and the functions are evaluated for a single step, the resulting values of the BNE deviate slightly from the values of the successor state of the corresponding BN. The BNE functions reflect the Boolean rules of the Boolean Network which regulate the expression of genes. The BNE models the influence of genes on other genes in a continuous manner. The resulting values of BNE can be seen as propensity for gene expression. As genes interact with each other via the concentration of gene products, a propensity is similar to concentration levels.

Similarly to the fixed points of the BN, we relate the resulting fixed points to biological phenotypes. We use a distance based approach to map fixed points to phenotypes. In order to compare the fixed points we compute Euclidean distances to the biological phenotypes. The hypothetical phenotype which has the shortest distance to a fixed point corresponds to the phenotype of the fixed point. In case of perfect agreement between the fixed point and the phenotype the distance is 0. In order to find the best corresponding phenotype to a fixed point it is necessary to exhaustively test the set of all hypothetical phenotypes.

Since the values of genes of the fixed points represent only gene expression propensities, one could choose a threshold for which a certain gene propensity becomes either an activation or inactivation of a gene. However, this requires additional knowledge about the property of the gene. Usually, the threshold is not easily determined and the choice may be arbitrary. We avoid choosing a threshold by using the proposed distance based approach.

The fixed points of the BNE for the cardiac development are parametrized by the *exogen*_*canWnt*_*I* parameter. This parameter influences all core genes of the model except for exogenous Bmp2 parameters. This means that the fixed points of the cardiac model are not just single points, but the values of the fixed point are continuous curves that depend directly on the gene expression propensity of the exogenous canonical Wnt. Further, the cardiac model directly encodes the *exogen*_*Bmp*2_*I* as a constant in the model. If we allow interval values for the *exogen*_*Bmp*2_*I*, it would directly influence the interaction of the core genes. This would result in fixed points that depend on two independent parameters and form a plane of fixed points. Here, however, we want to remain faithful to the original Boolean model for cardiac development where the *exogen*_*Bmp*2_*I* parameter is needed to be “on” in order to start the FHF and SHF formation [40].

The additional phenotypes, that are described by the fixed points of the BNE, are not found in the BN due to its discrete nature. However, these phenotypes are found to be biologically relevant to the early cardiac development. In general, the examination of BN attractors in perturbative manner with the BNE makes it possible to further characterize any Boolean model. The attractors of the cardiac development BN model are per definition fixed points in the BNE. In case of the 1% attractor from the Boolean Model, we found that this fixed point is unstable. Any perturbation of this fixed point leads to the FHF phenotype of the BN.

In our approach we use the nearest neighbor method to map fixed points to phenotypes. We do so by only considering a subset of genes that are know from the literature. However, once we have mapped the phenotypes, we can explore genes for which no information is available. The gene expression propensity of the additional genes is given by the fixed points of the simulated model. We thus can predict what a phenotype would look like by inspecting the complete set of genes for the nearest hypothetical phenotype as has been shown in the simulation of the BNE for the 11 core genes for early cardiac development.

## Supporting Information

S1 Supplementary Information File Detailed description of the cardiac and cell cycle BN and BNE. Additional information regarding parametric dependency and biological relevance for the subset of modelled genes, simulations with varying *exogen*_*Bmp*2_*I* parameter, and min–max operator.
(PDF)
