# Supplementary material for: Predicting Variabilities in Cardiac Gene Expression with a Boolean Network Incorporating Uncertainty
Source: PLoS One. 2015 Jul 24;10(7):e0131832. doi: 10.1371/journal.pone.0131832 (PMC4514755; doi:10.1371/journal.pone.0131832)
Supplement: S1 Supplementary Information File — Additional information regarding parametric dependency and biological relevance for the subset of modelled genes, simulations with varying exogen_Bmp2_I parameter, and min–max operator. (PDF) [file pone.0131832.s001.pdf]

Predicting variabilities in cardiac gene expression with a Boolean network incorporating uncertainty

Supplementary Information

Melanie Grieb[2,5,*], Andre Burkovski[2,3,5,*], J. Eric Sträng[2,*], Johann M. Kraus[2], Alexander Groß[2], Günther Palm[3], Michael Kühl[4,**], Hans A. Kestler[1,2,3,**]

1 Leibniz Institute for Age Research, Fritz-Lipmann Institute, 07745 Jena, Germany
2 Core Unit Medical Systems Biology, Ulm University, 89069 Ulm, Germany
3 Neural Information Processing, Ulm University, 89069 Ulm, Germany
4 Institute for Biochemistry and Molecular Biology, Ulm University, 89069 Ulm, Germany
5 International Graduate School in Molecular Medicine, Ulm University, 89069 Ulm, Germany
∗ equal contribution
∗∗ corresponding authors email: michael.kuehl@uni-ulm.de, hans.kestler@uni-ulm.de, hkestler@fli-leibniz.de

A Boolean network model of cardiac network

The original Boolean network model of the cardiac network is presented in Herrmann et al. [1]. Table A shows the Boolean rules that describe the interactions between the genes. Fig. A shows the network wiring of the cardiac development model.

Table A. Boolean network model of cardiac network: Boolean formulae for the state transitions. The genes of the network are shown in the left column. The subsequent state of each gene is constructed by applying the Boolean function in the right column to the previous state of the genes. Elementary Boolean functions are denoted by ¬ = NEGATION, ∨ = OR and ∧ = AND. The meaning of the variables can be found in Table 1 in the main text.

**t+1**	**t**
**Intracellular factors**	
$Bmp2$	$\neg canWnt \wedge exogen\_BMP2\_II$
$canWnt$	$exogen\_CanWnt\_II$
$Dkk1$	$Mesp1 \vee (canWnt \wedge \neg exogen\_BMP2\_II)$
$Fgf8$	$\neg Mesp1 \wedge (Foxc1.2 \vee Tbx1)$
$Foxc1.2$	$canWnt \wedge exogen\_CanWnt\_II$
$GATAs$	$Nkx2.5 \vee Mesp1 \vee Tbx5$
$Isl1$	$Tbx1 \vee Mesp1 \vee Fgf8 \vee (canWnt \wedge exogen\_CanWnt\_II)$
$Mesp1$	$canWnt \wedge exogen\_BMP2\_II$
$Nkx2.5$	$(Isl1 \wedge GATAs) \vee Tbx1 \vee (Mesp1 \wedge Dkk1) \vee (Bmp2 \wedge GATAs) \vee Tbx5$
$Tbx1$	$Foxc1.2$
$Tbx5$	$\neg(Tbx1 \vee canWnt) \wedge (Nkx2.5 \vee Tbx5 \vee Mesp1) \wedge \neg(Dkk1 \wedge \neg(Mesp1 \vee Tbx5)$
**Extracellular factors**	
$exogen\_BMP2\_I$	1
$exogen\_BMP2\_II$	$exogen\_BMP2\_I$
$exogen\_CanWnt\_I$	$exogen\_CanWnt\_I$
$exogen\_CanWnt\_II$	$exogen\_CanWnt\_I$

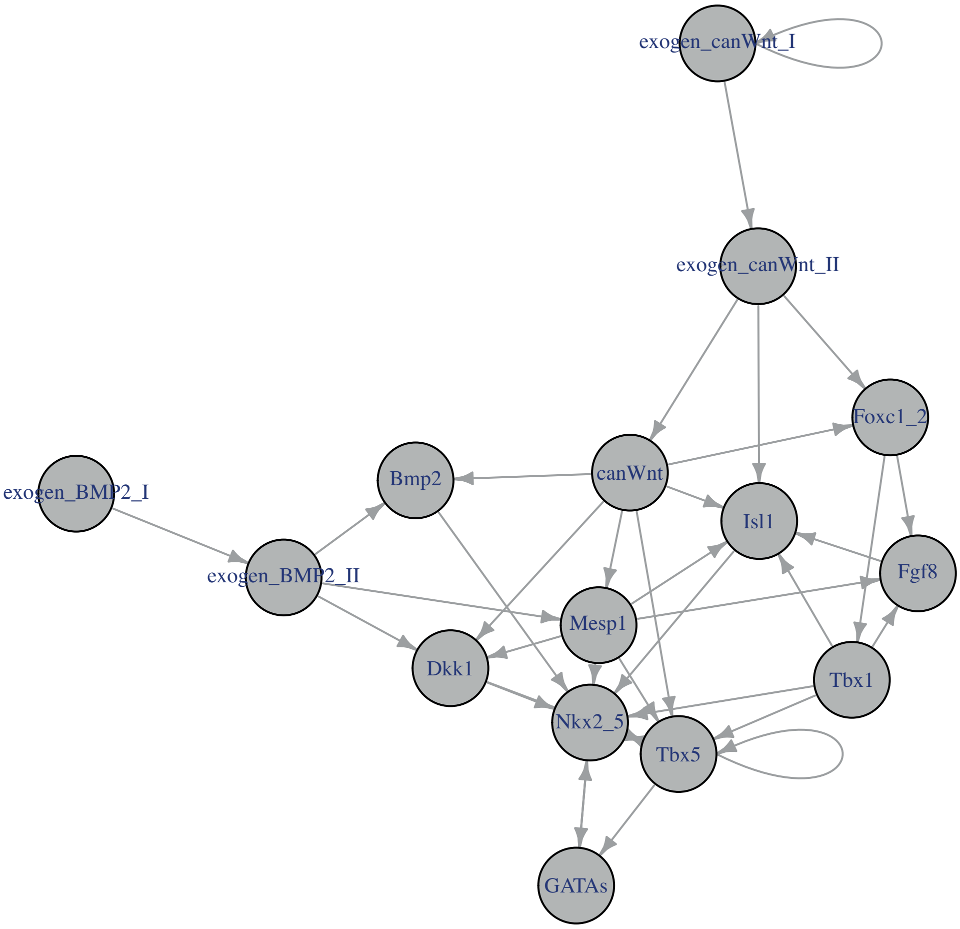

Fig. A. Cardiac Development network. The figure shows the network wiring for the Boolean network. The plot was created using BoolNet package [2].

B Boolean Network Extension of cardiac network

Table B shows the rules of the Boolean Network Extension. In the extension, Boolean rules are reformulated to their equivalent Disjunctive Normal Form and are then extended by applying product-sum fuzzy logic transformation as explained in the main text.

Table B. Boolean network extension of cardiac network: resulting polynomials for the state transitions. The genes of the network are shown in the left column of the table. The subsequent value of each gene is computed by the polynomial function shown in the right column. The meaning of the variables can be found in Table 1 in the main text.

**t+1**	**t**
$Bmp2$	$((1 - canWnt) \cdot exogen\_BMP2\_II)$
$canWnt$	$exogen\_canWnt\_II$
$Dkk1$	$((1 - canWnt) \cdot Mesp1 \cdot (1 - exogen\_BMP2\_II)) + ((1 - canWnt) \cdot Mesp1 \cdot exogen\_BMP2\_II) + (canWnt \cdot (1 - Mesp1) \cdot (1 - exogen\_BMP2\_II)) + (canWnt \cdot Mesp1 \cdot (1 - exogen\_BMP2\_II)) + (canWnt \cdot Mesp1 \cdot exogen\_BMP2\_II)$
$Fgf8$	$((1 - Foxc1\_2) \cdot (1 - Mesp1) \cdot Tbx1) + (Foxc1\_2 \cdot (1 - Mesp1) \cdot (1 - Tbx1)) + (Foxc1\_2 \cdot (1 - Mesp1) \cdot Tbx1)$
$Foxc1.2$	$(canWnt \cdot exogen\_canWnt\_II)$
$GATAs$	$((1 - Mesp1) \cdot (1 - Nkx2.5) \cdot Tbx5) + ((1 - Mesp1) \cdot Nkx2.5 \cdot (1 - Tbx5)) + ((1 - Mesp1) \cdot Nkx2.5 \cdot Tbx5) + (Mesp1 \cdot (1 - Nkx2.5) \cdot (1 - Tbx5)) + (Mesp1 \cdot (1 - Nkx2.5) \cdot Tbx5) + (Mesp1 \cdot Nkx2.5 \cdot (1 - Tbx5)) + (Mesp1 \cdot Nkx2.5 \cdot Tbx5)$
$Isl1$	$((1 - canWnt) \cdot (1 - Fgf8) \cdot (1 - Mesp1) \cdot Tbx1 \cdot (1 - exogen\_canWnt\_II)) + ((1 - canWnt) \cdot (1 - Fgf8) \cdot (1 - Mesp1) \cdot Tbx1 \cdot exogen\_canWnt\_II) + ((1 - canWnt) \cdot (1 - Fgf8) \cdot Mesp1 \cdot (1 - Tbx1) \cdot (1 - exogen\_canWnt\_II)) + ((1 - canWnt) \cdot (1 - Fgf8) \cdot Mesp1 \cdot (1 - Tbx1) \cdot exogen\_canWnt\_II) + ((1 - canWnt) \cdot (1 - Fgf8) \cdot Mesp1 \cdot Tbx1 \cdot (1 - exogen\_canWnt\_II)) + ((1 - canWnt) \cdot (1 - Fgf8) \cdot Mesp1 \cdot Tbx1 \cdot exogen\_canWnt\_II) + ((1 - canWnt) \cdot Fgf8 \cdot (1 - Mesp1) \cdot (1 - Tbx1) \cdot (1 - exogen\_canWnt\_II)) + ((1 - canWnt) \cdot Fgf8 \cdot (1 - Mesp1) \cdot (1 - Tbx1) \cdot exogen\_canWnt\_II) + ((1 - canWnt) \cdot Fgf8 \cdot (1 - Mesp1) \cdot Tbx1 \cdot (1 - exogen\_canWnt\_II)) + ((1 - canWnt) \cdot Fgf8 \cdot (1 - Mesp1) \cdot Tbx1 \cdot exogen\_canWnt\_II) + ((1 - canWnt) \cdot Fgf8 \cdot Mesp1 \cdot (1 - Tbx1) \cdot (1 - exogen\_canWnt\_II)) + ((1 - canWnt) \cdot Fgf8 \cdot Mesp1 \cdot (1 - Tbx1) \cdot exogen\_canWnt\_II) + ((1 - canWnt) \cdot Fgf8 \cdot Mesp1 \cdot Tbx1 \cdot (1 - exogen\_canWnt\_II)) + ((1 - canWnt) \cdot Fgf8 \cdot Mesp1 \cdot Tbx1 \cdot exogen\_canWnt\_II) + (canWnt \cdot (1 - Fgf8) \cdot (1 - Mesp1) \cdot (1 - Tbx1) \cdot exogen\_canWnt\_II) + (canWnt \cdot (1 - Fgf8) \cdot (1 - Mesp1) \cdot Tbx1 \cdot (1 - exogen\_canWnt\_II)) + (canWnt \cdot (1 - Fgf8) \cdot (1 - Mesp1) \cdot Tbx1 \cdot exogen\_canWnt\_II) + (canWnt \cdot (1 - Fgf8) \cdot Mesp1 \cdot (1 - Tbx1) \cdot (1 - exogen\_canWnt\_II)) + (canWnt \cdot (1 - Fgf8) \cdot Mesp1 \cdot (1 - Tbx1) \cdot exogen\_canWnt\_II) + (canWnt \cdot (1 - Fgf8) \cdot Mesp1 \cdot Tbx1 \cdot (1 - exogen\_canWnt\_II)) + (canWnt \cdot (1 - Fgf8) \cdot Mesp1 \cdot Tbx1 \cdot exogen\_canWnt\_II) + (canWnt \cdot Fgf8 \cdot (1 - Mesp1) \cdot (1 - Tbx1) \cdot (1 - exogen\_canWnt\_II)) + (canWnt \cdot Fgf8 \cdot (1 - Mesp1) \cdot (1 - Tbx1) \cdot exogen\_canWnt\_II) + (canWnt \cdot Fgf8 \cdot (1 - Mesp1) \cdot Tbx1 \cdot (1 - exogen\_canWnt\_II)) + (canWnt \cdot Fgf8 \cdot (1 - Mesp1) \cdot Tbx1 \cdot exogen\_canWnt\_II) + (canWnt \cdot Fgf8 \cdot Mesp1 \cdot (1 - Tbx1) \cdot (1 - exogen\_canWnt\_II)) + (canWnt \cdot Fgf8 \cdot Mesp1 \cdot (1 - Tbx1) \cdot exogen\_canWnt\_II) + (canWnt \cdot Fgf8 \cdot Mesp1 \cdot Tbx1 \cdot (1 - exogen\_canWnt\_II)) + (canWnt \cdot Fgf8 \cdot Mesp1 \cdot Tbx1 \cdot exogen\_canWnt\_II)$
$Mesp1$	$(canWnt \cdot (1 - exogen\_BMP2\_II))$

$Nkx2.5$	$((1-Bmp2)\cdot(1-Dkk1)\cdot(1-GATAs)\cdot(1-Isl1)\cdot(1-Mesp1)\cdot(1-Tbx1)\cdot Tbx5)+((1-Bmp2)\cdot(1-Dkk1)\cdot(1-GATAs)\cdot(1-Isl1)\cdot(1-Mesp1)\cdot Tbx1\cdot(1-Tbx5))+((1-Bmp2)\cdot(1-Dkk1)\cdot(1-GATAs)\cdot(1-Isl1)\cdot(1-Mesp1)\cdot Tbx1\cdot Tbx5)+((1-Bmp2)\cdot(1-Dkk1)\cdot(1-GATAs)\cdot(1-Isl1)\cdot Mesp1\cdot(1-Tbx1)\cdot Tbx5)+((1-Bmp2)\cdot(1-Dkk1)\cdot(1-GATAs)\cdot(1-Isl1)\cdot Mesp1\cdot Tbx1\cdot(1-Tbx5))+((1-Bmp2)\cdot(1-Dkk1)\cdot(1-GATAs)\cdot(1-Isl1)\cdot Mesp1\cdot Tbx1\cdot Tbx5)+((1-Bmp2)\cdot(1-Dkk1)\cdot(1-GATAs)\cdot Isl1\cdot(1-Mesp1)\cdot(1-Tbx1)\cdot Tbx5)+((1-Bmp2)\cdot(1-Dkk1)\cdot(1-GATAs)\cdot Isl1\cdot(1-Mesp1)\cdot Tbx1\cdot(1-Tbx5))+((1-Bmp2)\cdot(1-Dkk1)\cdot(1-GATAs)\cdot Isl1\cdot(1-Mesp1)\cdot Tbx1\cdot Tbx5)+((1-Bmp2)\cdot(1-Dkk1)\cdot(1-GATAs)\cdot Isl1\cdot Mesp1\cdot(1-Tbx1)\cdot Tbx5)+((1-Bmp2)\cdot(1-Dkk1)\cdot(1-GATAs)\cdot Isl1\cdot Mesp1\cdot Tbx1\cdot(1-Tbx5))+((1-Bmp2)\cdot(1-Dkk1)\cdot(1-GATAs)\cdot Isl1\cdot Mesp1\cdot Tbx1\cdot Tbx5)+((1-Bmp2)\cdot(1-Dkk1)\cdot GATAs\cdot(1-Isl1)\cdot(1-Mesp1)\cdot(1-Tbx1)\cdot Tbx5)+((1-Bmp2)\cdot(1-Dkk1)\cdot GATAs\cdot(1-Isl1)\cdot(1-Mesp1)\cdot Tbx1\cdot(1-Tbx5))+((1-Bmp2)\cdot(1-Dkk1)\cdot GATAs\cdot(1-Isl1)\cdot(1-Mesp1)\cdot Tbx1\cdot Tbx5)+((1-Bmp2)\cdot(1-Dkk1)\cdot GATAs\cdot(1-Isl1)\cdot Mesp1\cdot(1-Tbx1)\cdot Tbx5)+((1-Bmp2)\cdot(1-Dkk1)\cdot GATAs\cdot(1-Isl1)\cdot Mesp1\cdot Tbx1\cdot(1-Tbx5))+((1-Bmp2)\cdot(1-Dkk1)\cdot GATAs\cdot(1-Isl1)\cdot Mesp1\cdot Tbx1\cdot Tbx5)+((1-Bmp2)\cdot(1-Dkk1)\cdot GATAs\cdot Isl1\cdot(1-Mesp1)\cdot(1-Tbx1)\cdot(1-Tbx5))+((1-Bmp2)\cdot(1-Dkk1)\cdot GATAs\cdot Isl1\cdot(1-Mesp1)\cdot(1-Tbx1)\cdot Tbx5)+((1-Bmp2)\cdot(1-Dkk1)\cdot GATAs\cdot Isl1\cdot(1-Mesp1)\cdot Tbx1\cdot(1-Tbx5))+((1-Bmp2)\cdot(1-Dkk1)\cdot GATAs\cdot Isl1\cdot(1-Mesp1)\cdot Tbx1\cdot Tbx5)+((1-Bmp2)\cdot(1-Dkk1)\cdot GATAs\cdot Isl1\cdot Mesp1\cdot(1-Tbx1)\cdot(1-Tbx5))+((1-Bmp2)\cdot(1-Dkk1)\cdot GATAs\cdot Isl1\cdot Mesp1\cdot(1-Tbx1)\cdot Tbx5)+((1-Bmp2)\cdot(1-Dkk1)\cdot GATAs\cdot Isl1\cdot Mesp1\cdot Tbx1\cdot(1-Tbx5))+((1-Bmp2)\cdot(1-Dkk1)\cdot GATAs\cdot Isl1\cdot Mesp1\cdot Tbx1\cdot Tbx5)+((1-Bmp2)\cdot Dkk1\cdot(1-GATAs)\cdot(1-Isl1)\cdot(1-Mesp1)\cdot(1-Tbx1)\cdot Tbx5)+((1-Bmp2)\cdot Dkk1\cdot(1-GATAs)\cdot(1-Isl1)\cdot(1-Mesp1)\cdot Tbx1\cdot(1-Tbx5))+((1-Bmp2)\cdot Dkk1\cdot(1-GATAs)\cdot(1-Isl1)\cdot(1-Mesp1)\cdot Tbx1\cdot Tbx5)+((1-Bmp2)\cdot Dkk1\cdot(1-GATAs)\cdot(1-Isl1)\cdot Mesp1\cdot(1-Tbx1)\cdot(1-Tbx5))+((1-Bmp2)\cdot Dkk1\cdot(1-GATAs)\cdot(1-Isl1)\cdot Mesp1\cdot(1-Tbx1)\cdot Tbx5)+((1-Bmp2)\cdot Dkk1\cdot(1-GATAs)\cdot(1-Isl1)\cdot Mesp1\cdot Tbx1\cdot(1-Tbx5))+((1-Bmp2)\cdot Dkk1\cdot(1-GATAs)\cdot(1-Isl1)\cdot Mesp1\cdot Tbx1\cdot Tbx5)+((1-Bmp2)\cdot Dkk1\cdot(1-GATAs)\cdot Isl1\cdot(1-Mesp1)\cdot(1-Tbx1)\cdot Tbx5)+((1-Bmp2)\cdot Dkk1\cdot(1-GATAs)\cdot Isl1\cdot(1-Mesp1)\cdot Tbx1\cdot(1-Tbx5))+((1-Bmp2)\cdot Dkk1\cdot(1-GATAs)\cdot Isl1\cdot(1-Mesp1)\cdot Tbx1\cdot Tbx5)+((1-Bmp2)\cdot Dkk1\cdot(1-GATAs)\cdot Isl1\cdot Mesp1\cdot(1-Tbx1)\cdot(1-Tbx5))+((1-Bmp2)\cdot Dkk1\cdot(1-GATAs)\cdot Isl1\cdot Mesp1\cdot(1-Tbx1)\cdot Tbx5)+((1-Bmp2)\cdot Dkk1\cdot(1-GATAs)\cdot Isl1\cdot Mesp1\cdot Tbx1\cdot(1-Tbx5))+((1-Bmp2)\cdot Dkk1\cdot(1-GATAs)\cdot Isl1\cdot Mesp1\cdot Tbx1\cdot Tbx5)+((1-Bmp2)\cdot Dkk1\cdot GATAs\cdot(1-Isl1)\cdot(1-Mesp1)\cdot(1-Tbx1)\cdot Tbx5)+((1-Bmp2)\cdot Dkk1\cdot GATAs\cdot(1-Isl1)\cdot(1-Mesp1)\cdot Tbx1\cdot(1-Tbx5))+((1-Bmp2)\cdot Dkk1\cdot GATAs\cdot(1-Isl1)\cdot(1-Mesp1)\cdot Tbx1\cdot Tbx5)+((1-Bmp2)\cdot Dkk1\cdot GATAs\cdot(1-Isl1)\cdot Mesp1\cdot(1-Tbx1)\cdot(1-Tbx5))+((1-Bmp2)\cdot Dkk1\cdot GATAs\cdot(1-Isl1)\cdot Mesp1\cdot(1-Tbx1)\cdot Tbx5)+((1-Bmp2)\cdot Dkk1\cdot GATAs\cdot(1-Isl1)\cdot Mesp1\cdot Tbx1\cdot(1-Tbx5))+((1-Bmp2)\cdot Dkk1\cdot GATAs\cdot(1-Isl1)\cdot Mesp1\cdot Tbx1\cdot Tbx5)+((1-Bmp2)\cdot Dkk1\cdot GATAs\cdot Isl1\cdot(1-Mesp1)\cdot(1-Tbx1)\cdot(1-Tbx5))+((1-Bmp2)\cdot Dkk1\cdot GATAs\cdot Isl1\cdot(1-Mesp1)\cdot(1-Tbx1)\cdot Tbx5)+((1-Bmp2)\cdot Dkk1\cdot GATAs\cdot Isl1\cdot(1-Mesp1)\cdot Tbx1\cdot(1-Tbx5))+((1-Bmp2)\cdot Dkk1\cdot GATAs\cdot Isl1\cdot(1-Mesp1)\cdot Tbx1\cdot Tbx5)+((1-Bmp2)\cdot Dkk1\cdot GATAs\cdot Isl1\cdot Mesp1\cdot(1-Tbx1)\cdot(1-Tbx5))+((1-Bmp2)\cdot Dkk1\cdot GATAs\cdot Isl1\cdot Mesp1\cdot(1-Tbx1)\cdot Tbx5)+((1-Bmp2)\cdot Dkk1\cdot GATAs\cdot Isl1\cdot Mesp1\cdot Tbx1\cdot(1-Tbx5))+((1-Bmp2)\cdot Dkk1\cdot GATAs\cdot Isl1\cdot Mesp1\cdot Tbx1\cdot Tbx5)+(Bmp2\cdot(1-Dkk1)\cdot(1-GATAs)\cdot(1-Isl1)\cdot(1-Mesp1)\cdot(1-Tbx1)\cdot Tbx5)+(Bmp2\cdot(1-Dkk1)\cdot(1-GATAs)\cdot(1-Isl1)\cdot(1-Mesp1)\cdot Tbx1\cdot(1-Tbx5))+(Bmp2\cdot(1-Dkk1)\cdot(1-GATAs)\cdot(1-Isl1)\cdot(1-Mesp1)\cdot Tbx1\cdot Tbx5)+(Bmp2\cdot(1-Dkk1)\cdot(1-GATAs)\cdot(1-Isl1)\cdot Mesp1\cdot(1-Tbx1)\cdot Tbx5)+(Bmp2\cdot(1-Dkk1)\cdot(1-GATAs)\cdot(1-Isl1)\cdot Mesp1\cdot Tbx1\cdot(1-Tbx5))+(Bmp2\cdot(1-Dkk1)\cdot(1-GATAs)\cdot(1-Isl1)\cdot Mesp1\cdot Tbx1\cdot Tbx5)+(Bmp2\cdot(1-Dkk1)\cdot(1-GATAs)\cdot Isl1\cdot(1-Mesp1)\cdot(1-Tbx1)\cdot Tbx5)+(Bmp2\cdot(1-Dkk1)\cdot(1-GATAs)\cdot Isl1\cdot(1-Mesp1)\cdot Tbx1\cdot(1-Tbx5))+(Bmp2\cdot(1-Dkk1)\cdot(1-GATAs)\cdot Isl1\cdot(1-Mesp1)\cdot Tbx1\cdot Tbx5)+(Bmp2\cdot(1-Dkk1)\cdot(1-GATAs)\cdot Isl1\cdot Mesp1\cdot(1-Tbx1)\cdot Tbx5)+(Bmp2\cdot(1-Dkk1)\cdot(1-GATAs)\cdot Isl1\cdot Mesp1\cdot Tbx1\cdot(1-Tbx5))+(Bmp2\cdot(1-Dkk1)\cdot(1-GATAs)\cdot Isl1\cdot Mesp1\cdot Tbx1\cdot Tbx5)+(Bmp2\cdot(1-Dkk1)\cdot GATAs\cdot(1-Isl1)\cdot(1-Mesp1)\cdot(1-Tbx1)\cdot(1-Tbx5))+(Bmp2\cdot(1-Dkk1)\cdot GATAs\cdot(1-Isl1)\cdot(1-Mesp1)\cdot(1-Tbx1)\cdot Tbx5)+(Bmp2\cdot(1-Dkk1)\cdot GATAs\cdot(1-Isl1)\cdot(1-Mesp1)\cdot Tbx1\cdot(1-Tbx5))+(Bmp2\cdot(1-Dkk1)\cdot GATAs\cdot(1-Isl1)\cdot(1-Mesp1)\cdot Tbx1\cdot Tbx5)+(Bmp2\cdot(1-Dkk1)\cdot GATAs\cdot(1-Isl1)\cdot Mesp1\cdot(1-Tbx1)\cdot(1-Tbx5))+(Bmp2\cdot(1-Dkk1)\cdot GATAs\cdot(1-Isl1)\cdot Mesp1\cdot(1-Tbx1)\cdot Tbx5)+(Bmp2\cdot(1-Dkk1)\cdot GATAs\cdot(1-Isl1)\cdot Mesp1\cdot Tbx1\cdot(1-Tbx5))+(Bmp2\cdot(1-Dkk1)\cdot GATAs\cdot(1-Isl1)\cdot Mesp1\cdot Tbx1\cdot Tbx5)+(Bmp2\cdot(1-Dkk1)\cdot GATAs\cdot Isl1\cdot(1-Mesp1)\cdot(1-Tbx1)\cdot(1-Tbx5))+(Bmp2\cdot(1-Dkk1)\cdot GATAs\cdot Isl1\cdot(1-Mesp1)\cdot(1-Tbx1)\cdot Tbx5)+(Bmp2\cdot(1-Dkk1)\cdot GATAs\cdot Isl1\cdot(1-Mesp1)\cdot Tbx1\cdot(1-Tbx5))+(Bmp2\cdot(1-Dkk1)\cdot GATAs\cdot Isl1\cdot(1-Mesp1)\cdot Tbx1\cdot Tbx5)+(Bmp2\cdot(1-Dkk1)\cdot GATAs\cdot Isl1\cdot Mesp1\cdot(1-Tbx1)\cdot(1-Tbx5))+(Bmp2\cdot(1-Dkk1)\cdot GATAs\cdot Isl1\cdot Mesp1\cdot(1-Tbx1)\cdot Tbx5)+(Bmp2\cdot(1-Dkk1)\cdot GATAs\cdot Isl1\cdot Mesp1\cdot Tbx1\cdot(1-Tbx5))+(Bmp2\cdot(1-Dkk1)\cdot GATAs\cdot Isl1\cdot Mesp1\cdot Tbx1\cdot Tbx5)+(Bmp2\cdot Dkk1\cdot(1-GATAs)\cdot(1-Isl1)\cdot(1-Mesp1)\cdot(1-Tbx1)\cdot Tbx5)+(Bmp2\cdot Dkk1\cdot(1-GATAs)\cdot(1-Isl1)\cdot(1-Mesp1)\cdot Tbx1\cdot(1-Tbx5))+(Bmp2\cdot Dkk1\cdot(1-GATAs)\cdot(1-Isl1)\cdot(1-Mesp1)\cdot Tbx1\cdot Tbx5)+(Bmp2\cdot Dkk1\cdot(1-GATAs)\cdot(1-Isl1)\cdot Mesp1\cdot(1-Tbx1)\cdot(1-Tbx5))+(Bmp2\cdot Dkk1\cdot(1-GATAs)\cdot(1-Isl1)\cdot Mesp1\cdot(1-Tbx1)\cdot Tbx5)+(Bmp2\cdot Dkk1\cdot(1-GATAs)\cdot(1-Isl1)\cdot Mesp1\cdot Tbx1\cdot(1-Tbx5))+(Bmp2\cdot Dkk1\cdot(1-GATAs)\cdot(1-Isl1)\cdot Mesp1\cdot Tbx1\cdot Tbx5)+(Bmp2\cdot Dkk1\cdot(1-GATAs)\cdot Isl1\cdot(1-Mesp1)\cdot(1-Tbx1)\cdot Tbx5)+(Bmp2\cdot Dkk1\cdot(1-GATAs)\cdot Isl1\cdot(1-Mesp1)\cdot Tbx1\cdot(1-Tbx5))+(Bmp2\cdot Dkk1\cdot(1-GATAs)\cdot Isl1\cdot(1-Mesp1)\cdot Tbx1\cdot Tbx5)+(Bmp2\cdot Dkk1\cdot(1-GATAs)\cdot Isl1\cdot Mesp1\cdot(1-Tbx1)\cdot(1-Tbx5))+(Bmp2\cdot Dkk1\cdot(1-GATAs)\cdot Isl1\cdot Mesp1\cdot(1-Tbx1)\cdot Tbx5)+(Bmp2\cdot Dkk1\cdot(1-GATAs)\cdot Isl1\cdot Mesp1\cdot Tbx1\cdot(1-Tbx5))+(Bmp2\cdot Dkk1\cdot(1-GATAs)\cdot Isl1\cdot Mesp1\cdot Tbx1\cdot Tbx5)+(Bmp2\cdot Dkk1\cdot GATAs\cdot(1-Isl1)\cdot(1-Mesp1)\cdot(1-Tbx1)\cdot(1-Tbx5))+(Bmp2\cdot Dkk1\cdot GATAs\cdot(1-Isl1)\cdot(1-Mesp1)\cdot(1-Tbx1)\cdot Tbx5)+(Bmp2\cdot Dkk1\cdot GATAs\cdot(1-Isl1)\cdot(1-Mesp1)\cdot Tbx1\cdot(1-Tbx5))+(Bmp2\cdot Dkk1\cdot GATAs\cdot(1-Isl1)\cdot(1-Mesp1)\cdot Tbx1\cdot Tbx5)+(Bmp2\cdot Dkk1\cdot GATAs\cdot(1-Isl1)\cdot Mesp1\cdot(1-Tbx1)\cdot(1-Tbx5))+(Bmp2\cdot Dkk1\cdot GATAs\cdot(1-Isl1)\cdot Mesp1\cdot(1-Tbx1)\cdot Tbx5)+(Bmp2\cdot Dkk1\cdot GATAs\cdot(1-Isl1)\cdot Mesp1\cdot Tbx1\cdot(1-Tbx5))+(Bmp2\cdot Dkk1\cdot GATAs\cdot(1-Isl1)\cdot Mesp1\cdot Tbx1\cdot Tbx5)+(Bmp2\cdot Dkk1\cdot GATAs\cdot Isl1\cdot(1-Mesp1)\cdot(1-Tbx1)\cdot(1-Tbx5))+(Bmp2\cdot Dkk1\cdot GATAs\cdot Isl1\cdot(1-Mesp1)\cdot(1-Tbx1)\cdot Tbx5)+(Bmp2\cdot Dkk1\cdot GATAs\cdot Isl1\cdot(1-Mesp1)\cdot Tbx1\cdot(1-Tbx5))+(Bmp2\cdot Dkk1\cdot GATAs\cdot Isl1\cdot(1-Mesp1)\cdot Tbx1\cdot Tbx5)+(Bmp2\cdot Dkk1\cdot GATAs\cdot Isl1\cdot Mesp1\cdot(1-Tbx1)\cdot(1-Tbx5))+(Bmp2\cdot Dkk1\cdot GATAs\cdot Isl1\cdot Mesp1\cdot(1-Tbx1)\cdot Tbx5)+(Bmp2\cdot Dkk1\cdot GATAs\cdot Isl1\cdot Mesp1\cdot Tbx1\cdot(1-Tbx5))+(Bmp2\cdot Dkk1\cdot GATAs\cdot Isl1\cdot Mesp1\cdot Tbx1\cdot Tbx5)$
$Tbx1$	$(Foxc1.2)$

$Tbx5$	$((1-canWnt)\cdot(1-Dkk1)\cdot(1-Mesp1)\cdot(1-Nkx2.5)\cdot(1-Tbx1)\cdot Tbx5)+((1-canWnt)\cdot(1-Dkk1)\cdot(1-Mesp1)\cdot Nkx2.5\cdot(1-Tbx1)\cdot(1-Tbx5))+((1-canWnt)\cdot(1-Dkk1)\cdot(1-Mesp1)\cdot Nkx2.5\cdot(1-Tbx1)\cdot Tbx5)+((1-canWnt)\cdot(1-Dkk1)\cdot Mesp1\cdot(1-Nkx2.5)\cdot(1-Tbx1)\cdot(1-Tbx5))+((1-canWnt)\cdot(1-Dkk1)\cdot Mesp1\cdot(1-Nkx2.5)\cdot(1-Tbx1)\cdot Tbx5)+((1-canWnt)\cdot(1-Dkk1)\cdot Mesp1\cdot Nkx2.5\cdot(1-Tbx1)\cdot(1-Tbx5))+((1-canWnt)\cdot(1-Dkk1)\cdot Mesp1\cdot Nkx2.5\cdot(1-Tbx1)\cdot Tbx5)+((1-canWnt)\cdot Dkk1\cdot(1-Mesp1)\cdot(1-Nkx2.5)\cdot(1-Tbx1)\cdot Tbx5)+((1-canWnt)\cdot Dkk1\cdot(1-Mesp1)\cdot Nkx2.5\cdot(1-Tbx1)\cdot Tbx5)+((1-canWnt)\cdot Dkk1\cdot Mesp1\cdot(1-Nkx2.5)\cdot(1-Tbx1)\cdot(1-Tbx5))+((1-canWnt)\cdot Dkk1\cdot Mesp1\cdot(1-Nkx2.5)\cdot(1-Tbx1)\cdot Tbx5)+((1-canWnt)\cdot Dkk1\cdot Mesp1\cdot Nkx2.5\cdot(1-Tbx1)\cdot(1-Tbx5))+((1-canWnt)\cdot Dkk1\cdot Mesp1\cdot Nkx2.5\cdot(1-Tbx1)\cdot Tbx5)$
**Exogen factors**	
$exogen\_BMP2\_I$	1
$exogen\_BMP2\_II$	$exogen\_BMP2\_I$
$exogen\_CanWnt\_I$	$exogen\_CanWnt\_I$
$exogen\_CanWnt\_II$	$exogen\_CanWnt\_I$

C Parametric fixed points of the cardiac network

Fig. B shows the parametric dependency of the modelled genes on the *exogen_canWnt_I* parameter. The simulation of the BNE for 10^4 different initial values reveals a continuous change in gene expression propensity of the modelled genes in dependence of *exogen_canWnt_I*.

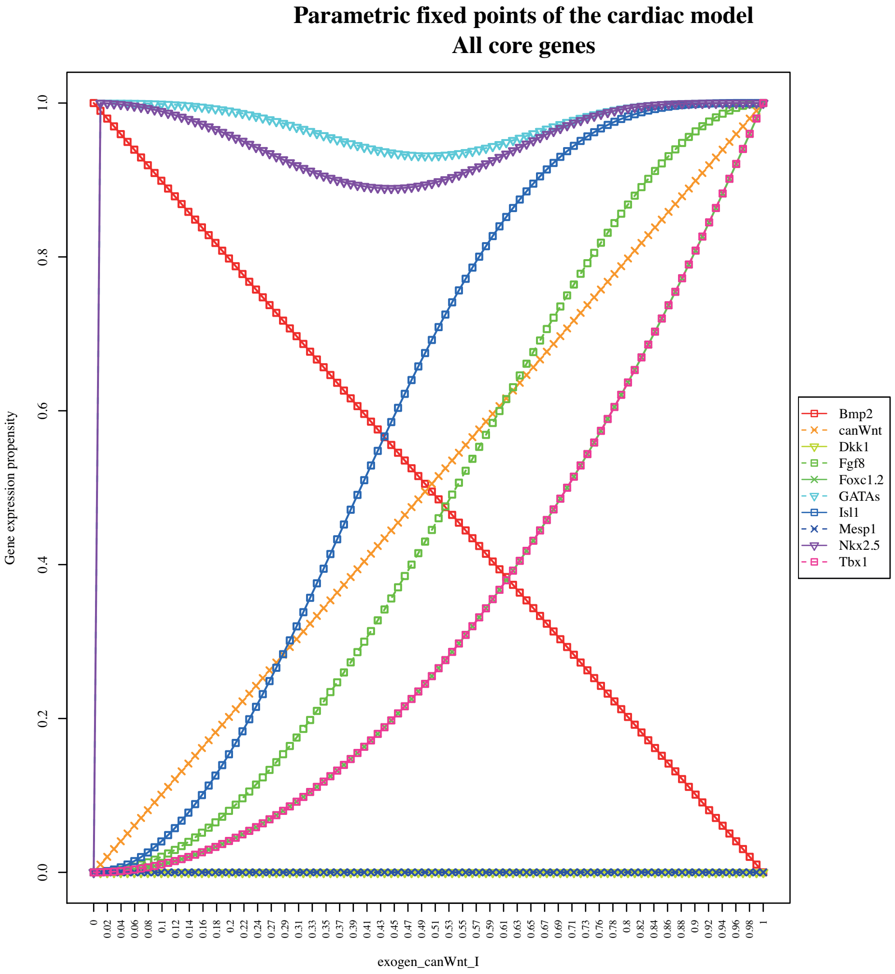

Fig. B. Dependency of fixed points on ***exogen_canWnt_I*** **parameter.** The x-axis shows the values of the parameter *exogen_canWnt_I*. The values for the fixed points of the model variables are shown on the y-axis. Each curve represents the fixed point value dependency of each individual gene. Setting all initial propensities to 0 results in the *NO_CARDIAC* fixed point in the Boolean Network. Otherwise, on the far ends (0 and 1) of *exogen_canWnt_I*, Boolean fixed points from the first heart field and second heart field are found. As gene expression propensities of *exogen_canWnt_I* changes additional intermediate fixed points are revealed that smoothly change their expression propensity in dependence of *exogen_canWnt_I*.

D Biological relevance for a subset of modelled genes.

We run simulation for the the 4 genes Isl1, Nkx2.5, Tbx1, Tbx5 that were analyzed in a RT-PCR in Gessert and Kühl [3]. The computed fixed points were then mapped to their nearest phenotypes as described in the main text. The 4 genes (Isl1, Nkx2.5, Tbx1, Tbx5) can give rise to $2^4 = 16$ different qualitative expression patterns. Five phenotypes are predicted by the BNE. In order to confirm the correspondence of the nearest phenotypes to the biological phenotypes we compared the gene expression propensity range for each gene in each phenotype (Fig. C). Starting from a point where no cardiac development takes place, the order of the phenotypes is FHF6, FHF7, SHF1 and SHF4 with increasing $exogen\_canWnt\_I$ expression propensity. The gene expression propensity of the SHF marker gene Isl1 is rather low for the FHF phenotype and is rather high for the SHF. In all mapped phenotypes, the Nkx2.5 expression propensity is rather high, which is also the case in the RT-PCR for the according phenotypes. The Tbx1 gene expression propensity is high for the SHF4 phenotype as well as Tbx5 for the FHF6 phenotype. The phenotype FHF7 continuously changes into the SHF1 phenotype based on the Isl1 expression propensity. Thus the BNE is able to identify biologically relevant phenotypes. Additionally we can state that the structure provided by the BN is sufficient to allow the prediction of these phenotypes through the extension.

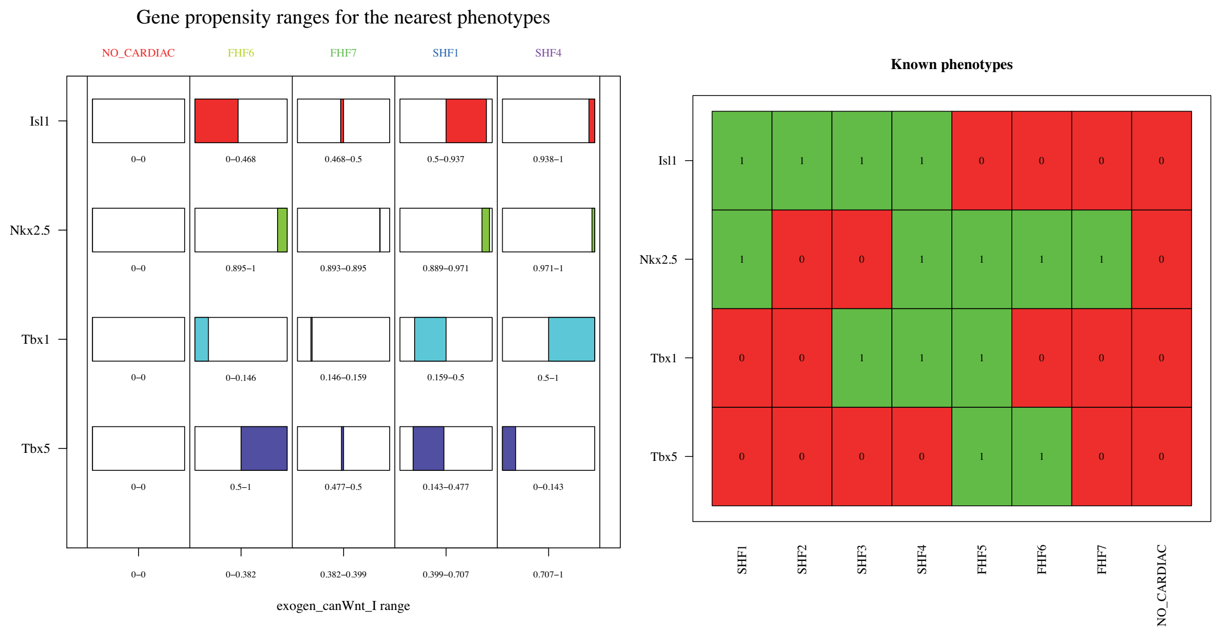

Fig. C. Propensity values for genes and phenotypes. The left figure shows the BNE gene expression propensity range for the 4 genes from a previous RT-PCR analysis that is schematically shown in the right figure [3]. Genes are shown on the left, phenotype names are shown on top. The range of the parameter $exogen\_canWnt\_I$ to each phenotype is shown at the bottom. The bars and numbers show the different gene expression propensity ranges per phenotype and gene. The gene expression propensities show a pattern (high/low) that is similar to the phenotypes from the RT-PCR analysis shown in the right figure (active/inactive).

E Variation of BMP2 input.

Supplementary Figs. E–H show the parametric dependency of the modelled genes on the *exogen_canWnt_I* and *exogen_Bmp2_I* parameter. The simulation of the BNE for different initial values for both parameters induces a continuous change in gene expression propensity. The mapping to phenotypes reveals more nearest phenotypes due to the change of *exogen_Bmp2_I* (Fig. D). The mapped expression propensity for the 4 core genes (Isl1, Nkx2.5, Tbx1, Tbx5) correspond to the phenotypes analyzed in a RT-PCR in Gessert and Kühl [3]. Due to the variability of *exogen_Bmp2_I* several new sub-phenotypes are predicted for each of the four main phenotypes (FHF6, FHF7, SHF1, and SHF4). In order to asses the predictive power of the model, these phenotypes need to be further confirmed in biological setting. Fig. I shows the propensity ranges for individual phenotypes. It is worth noting that some phenotypes are only identified though the propensity change of *exogen_canWnt_I* and *exogen_Bmp2_I*.

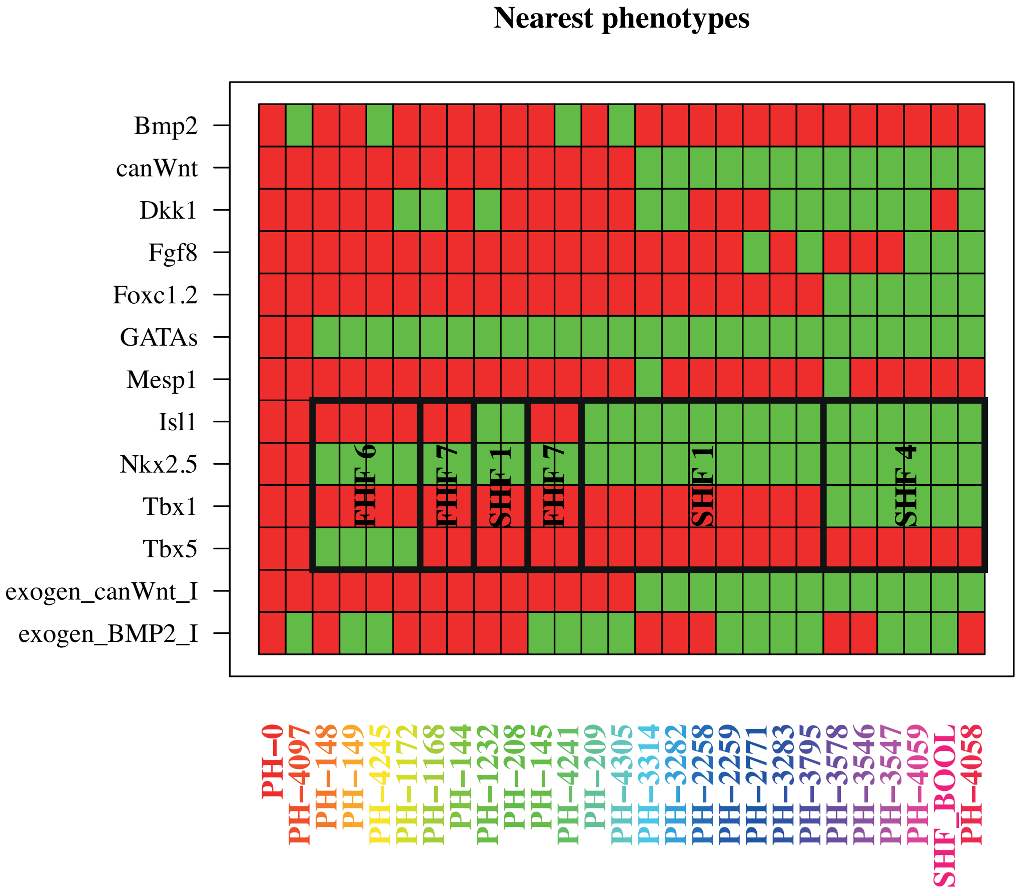

Fig. D. Mapped nearest phenotypes The figure shows the nearest phenotypes for the different fixed points found by for each pair of *exogen_canWnt_I* and *exogen_Bmp2_I* start value. The associated phenotypes here are plotted with no particular order.

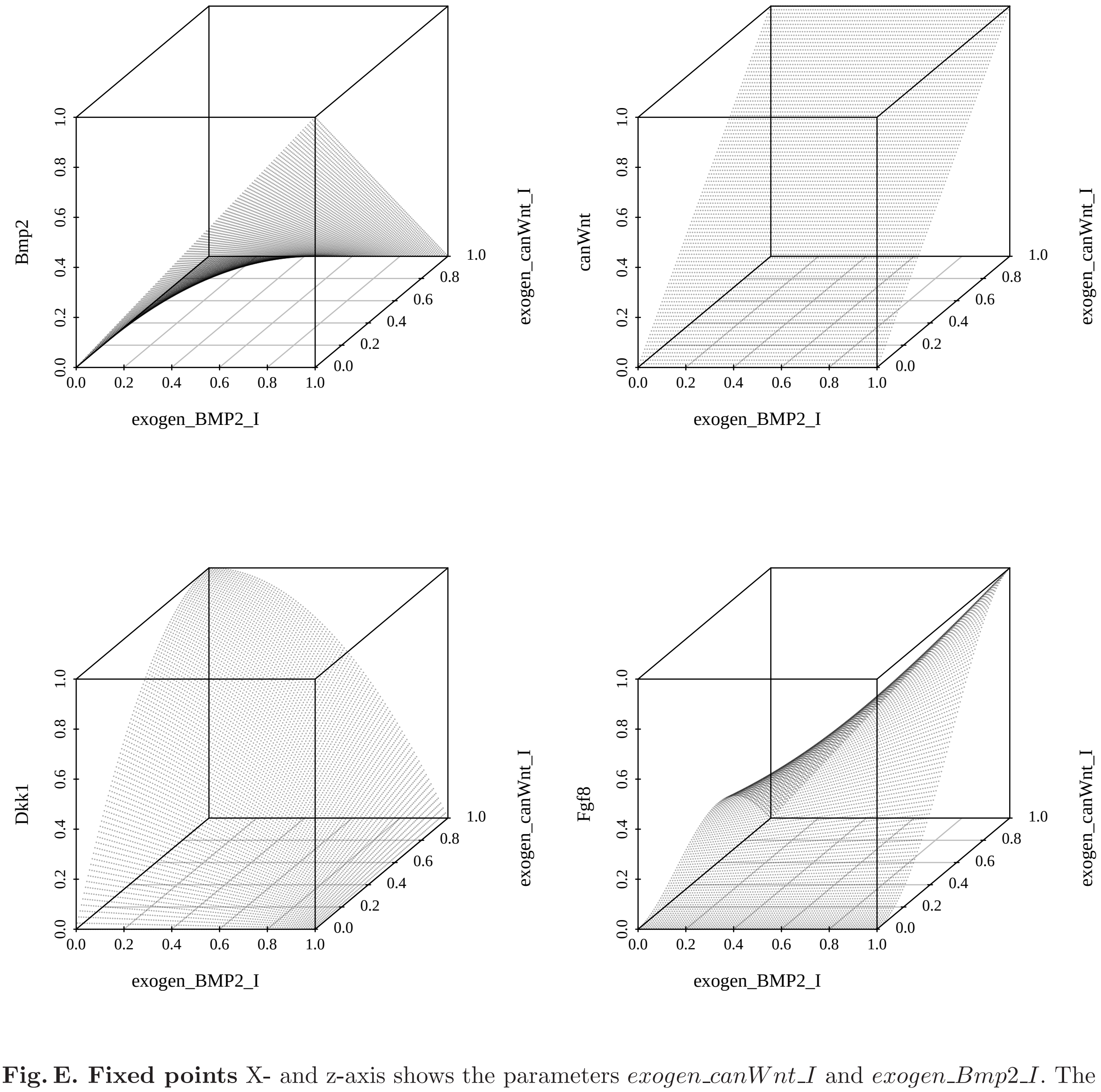

Fig. E. Fixed points X- and z-axis shows the parameters *exogen_canWnt_I* and *exogen_Bmp2_I*. The results for one of the input variables is shown on the y-axis. The fixed points lie on a 2-D plane.

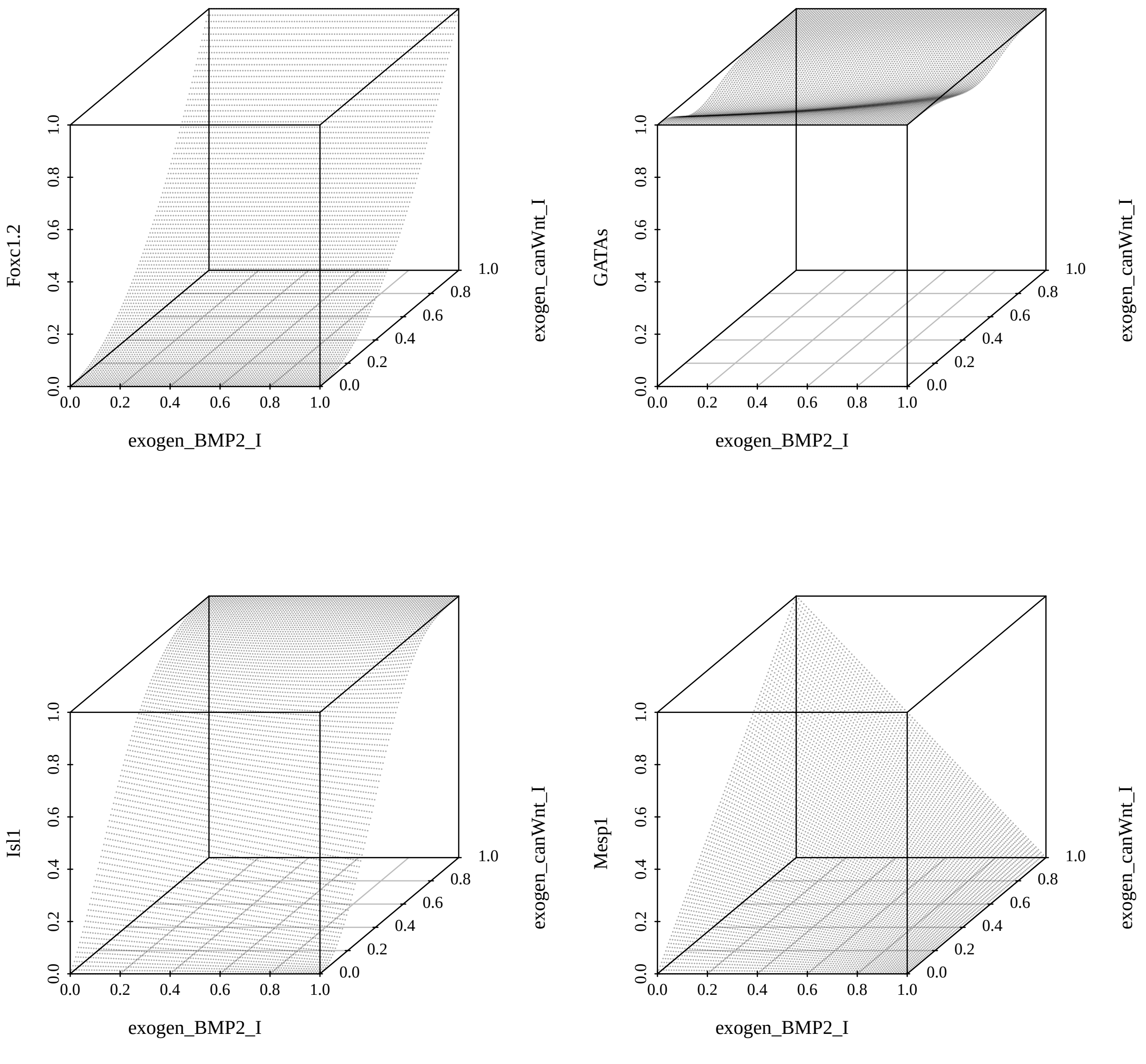

Fig. F. Fixed points X- and z-axis shows the parameters $exogen\_canWnt\_I$ and $exogen\_Bmp2\_I$. The results for one of the input variables is shown on the y-axis. The fixed points lie on a 2-D plane.

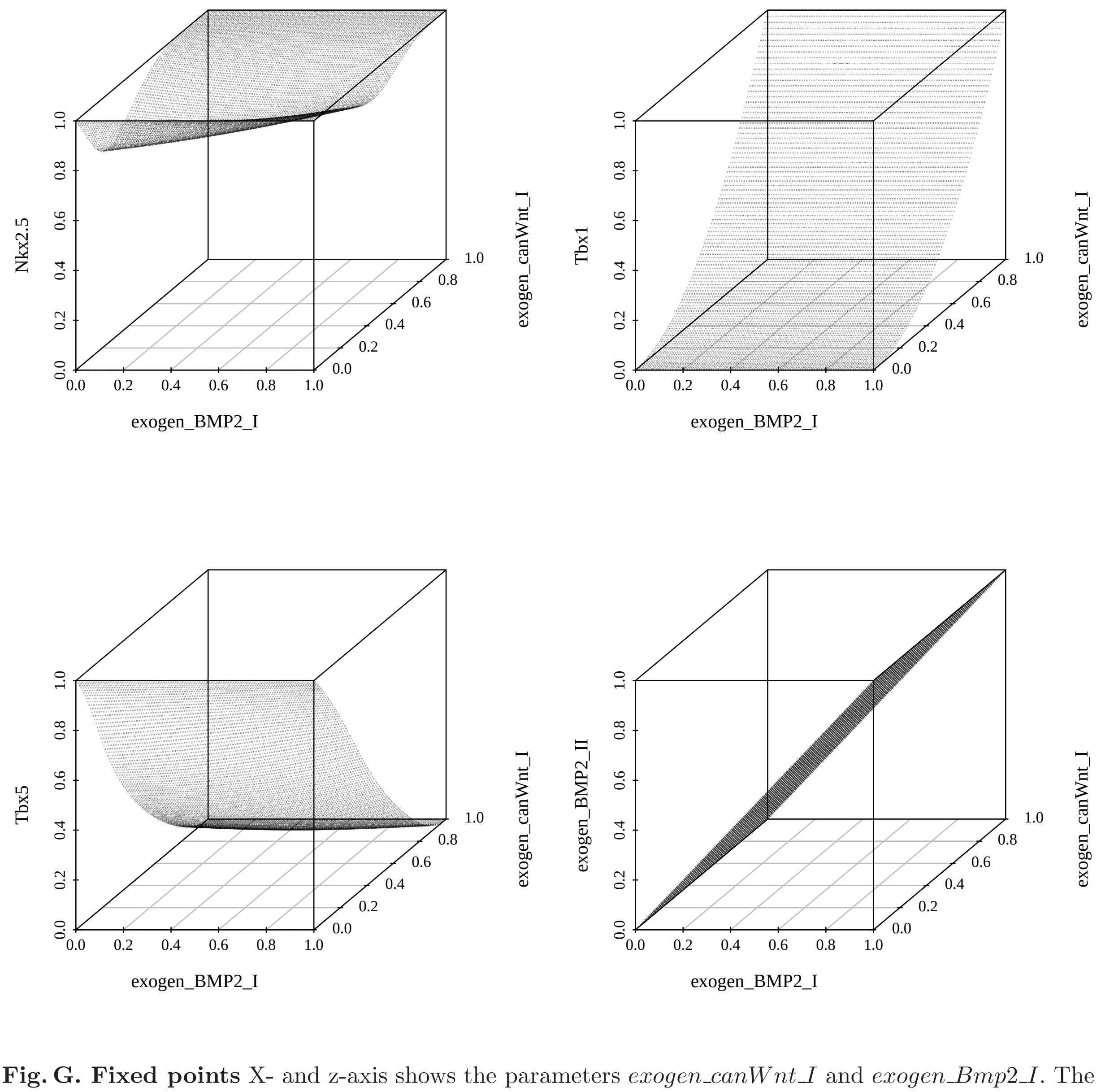

Fig. G. Fixed points X- and z-axis shows the parameters $exogen\_canWnt\_I$ and $exogen\_Bmp2\_I$. The results for one of the input variables is shown on the y-axis. The fixed points lie on a 2-D plane.

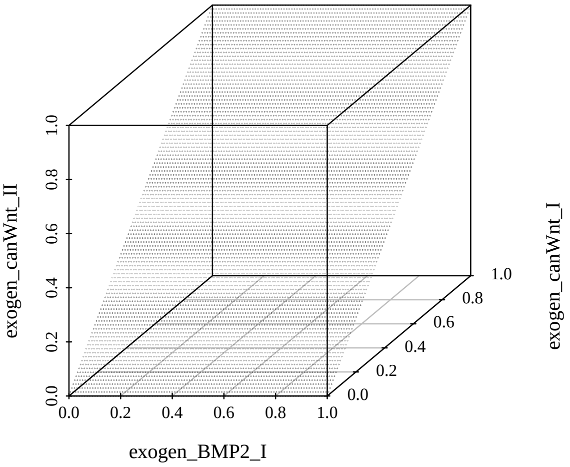

Fig. H. Fixed points X- and z-axis shows the parameters *exogen_canWnt_I* and *exogen_Bmp2_I*. The results for one of the input variables is shown on the y-axis. The fixed points lie on a 2-D plane.

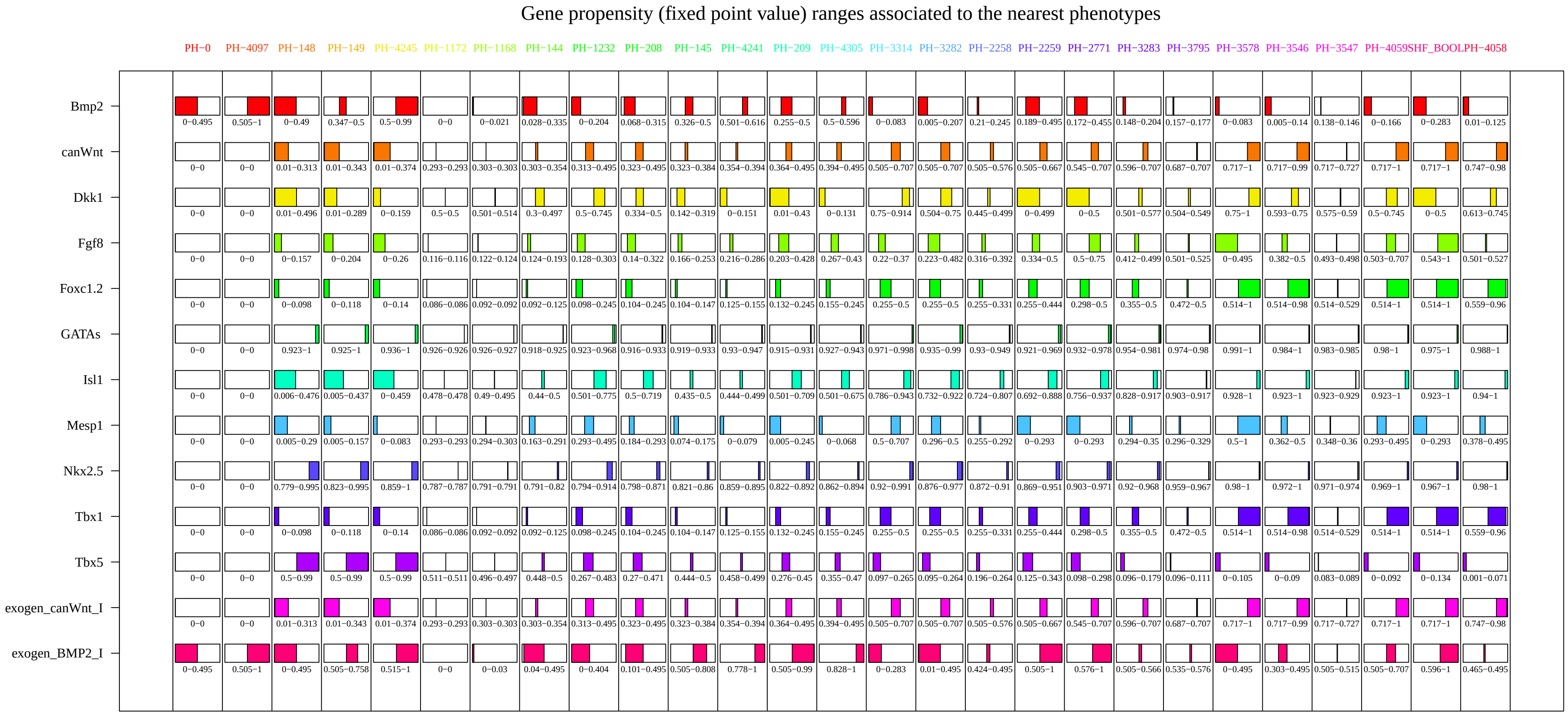

Fig. I. Gene propensity ranges The figure shows the gene propensity ranges for each of the phenotypes in supplementary Fig. D.

F Fixed points for min-max fuzzy logic.

The min–max fuzzy logic transforms the Boolean term in the following manner:

$$\widehat{\neg x_i} = 1 - \hat{x}_i,$$
$$\widehat{x_i \wedge x_j} = \min(\hat{x}_i, \hat{x}_j),$$
$$\widehat{x_i \vee x_j} = \max(\hat{x}_i, \hat{x}_j).$$

The resulting BNE was simulated using the iterative approach. We used only 100 random start values for linearly increasing *exogen_canWnt_I* we know since from previous simulations that the fixed point depend parametrically on *exogen_canWnt_I*. We discovered that the fixed points now not only depend on the *exogen_canWnt_I* parameter but also on the initial values of the variables. In particular, the variables *GATAs*, *Nkx*2.5, and *Tbx*5 have increased variation; all other variables remain dependent only on the *exogen_canWnt_I* parameter. Hence, we performed additional simulations for a fixed initial value of *exogen_canWnt_I* and 10000 random initial values for the remaining variables. Figs. J and K show examples for *exogen_canWnt_I* $\in \{0.25, 0.75\}$ and the resulting variances of *GATAs*, *Nkx*2.5, and *Tbx*5 variables in the fixed points. For *exogen_canWnt_I* < 0.5 the three variables have the same resulting propensity values (shown in the scatter plot). For *exogen_canWnt_I* > 0.5, *GATAs* and *Nkx*2.5 do not have a direct correlation and *Tbx*5 shows not variation. Fig. L shows a convergence graph for some initial values and *exogen_canWnt_I* $= 0.75$. The resulting trajectory converges to an attractor of length 2. Through the fact that oscillating behaviour occurs and that the oscillating values depend on the initial values of the simulation suggests that min–max approach is probably unsuited for the heart field model.

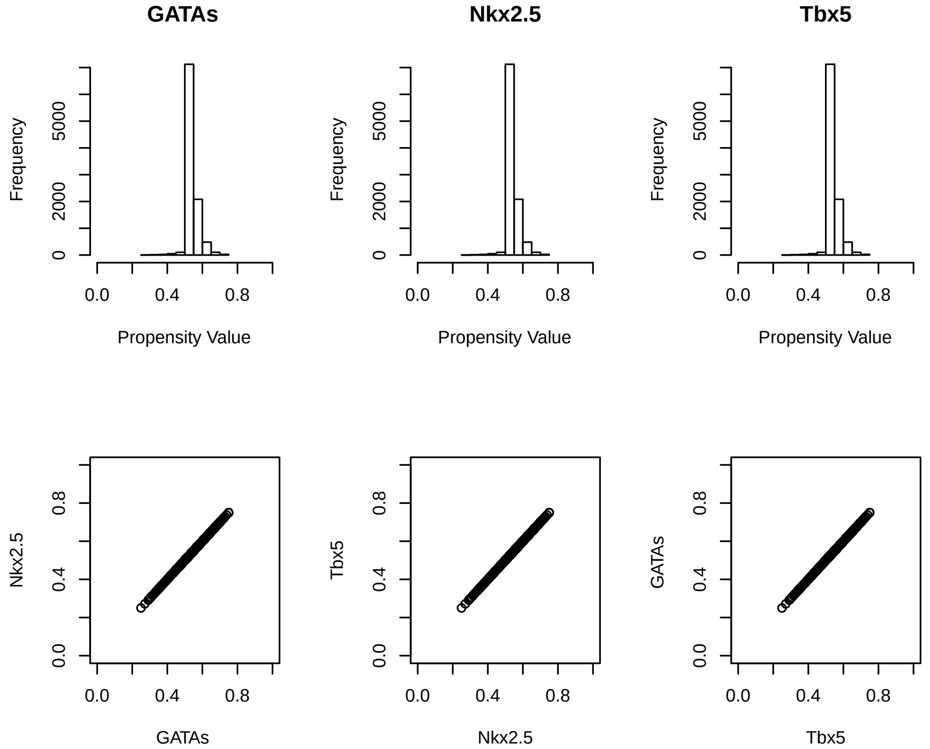

Fig. J. Variation of the values of the fixed points. The upper panel shows the variance of according variables for 10000 initial random values and *exogen_canWnt_I* $= 0.25$. The lower panel shows a scatter plot of the individual variables.

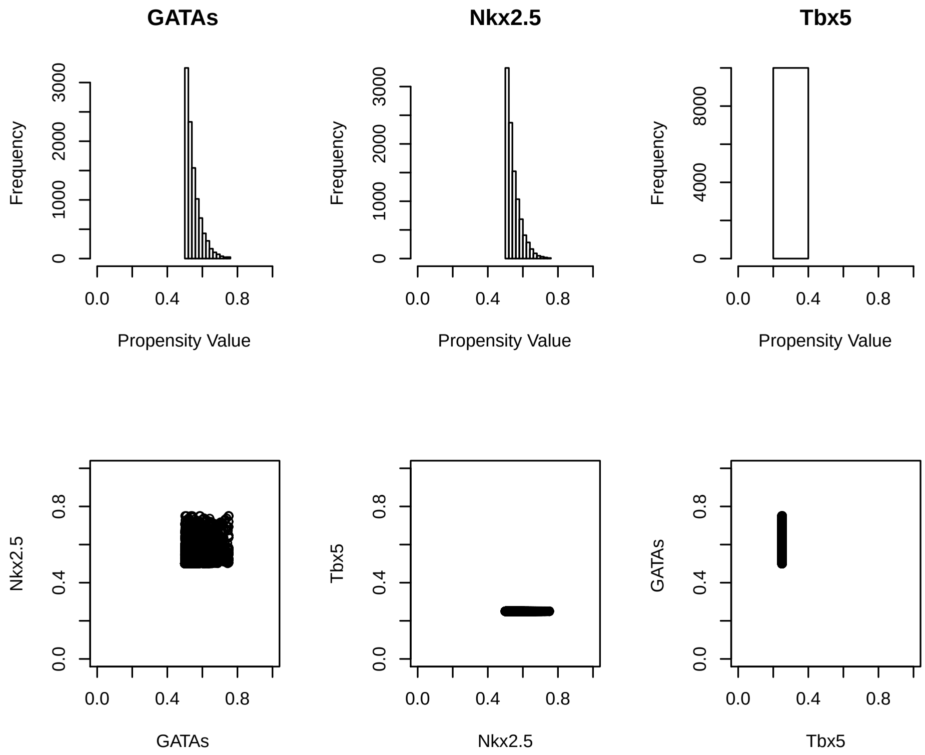

Fig. K. Variation of values in the fixed points. The upper panel shows the variance of according variables for 10000 initial random values and $exogen\_canWnt\_I = 0.75$. The lower panel shows a scatter plot of the individual variables.

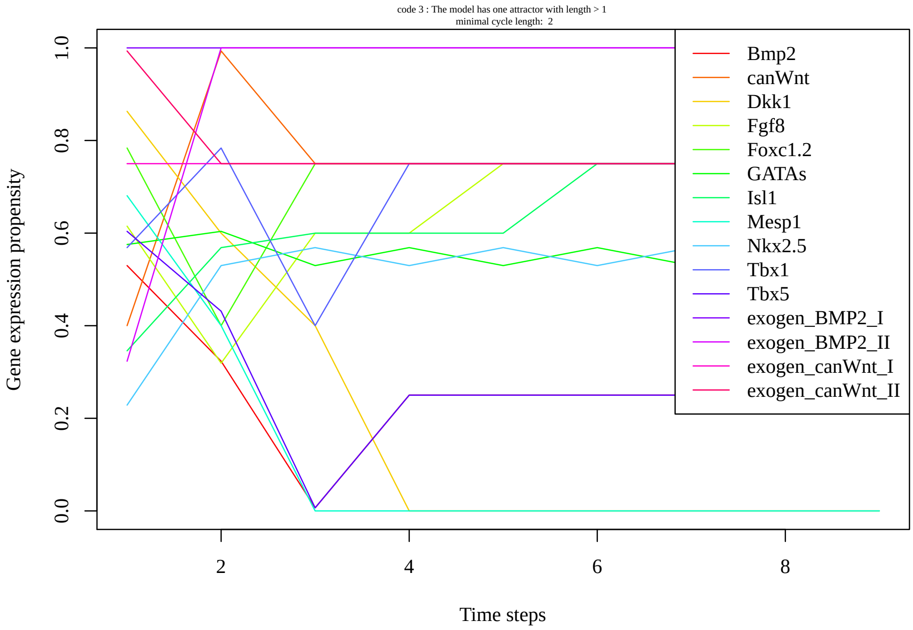

Fig. L. Convergence graph. The graph shows trajectories from initial values of the variables to the converged attractor ($exogen\_canWnt\_I = 0.75$). Oscillating behaviour can be seen for variables Nkx2.5 and GATAs.

G Mammalian Cell Cycle.

The gene regulatory network in Faure et al. [4] models the mammalian cell cycle in form of a Boolean Network (shown in Table C). It is derived from an ODE system described in Novak and Tyson [5]. The ODE model describes behavior of the cell cycle based on 27 equations and experimentally derived equation parameters. The Boolean Network [4] simplifies the ODE model by reducing the number of equations and focuses on interaction of Cyclins (CycA, CycB, CycD, CycE), Rb as representative gene for regulation of pRB, E2F as representation of unphosphorylated E2F products, p27, products from the APC complex Cdc20 and Cdh1, and UbcH10, a component not described in the original ODE. According to Faure et al. UcH10 is necessary for consistent results regarding CycA activation. The Boolean network models the state of a cell given CycD as an input variable and has two attractors: the first attractor has length 1 and represents the G0 phase of the cell, the second attractor has length 7 that represents the G1/S/G2/M phase.

We extend the BN of the mammalian cell cycle and determine the fixed points given the input variable CycD. Fig. M shows the parametric fixed points. In contrary to the time-independent cardiac phenotypes described in the main text, this model describes time-dependent molecular cell states. The fixed points of the BNE for mammalian cell cycle describe the molecular propensities according to CycD. Here, as in Faure, CycD is thought to evolve through the cell cycle and serves as a surrogate for overall time-dependence. The BNE system's response to CycD is approximated as instantaneous and fixed points hence are though to reflect the cell state. In effect the time is merely reflected by the changes in CycD. Fig. N shows the nearest molecular cell states that are mapped using the approach described in the main text. The cell states are ordered according increasing CycD propensity of the fixed points.

Since BNE is indirectly deduced from an ODE system, we expect the BNE to reproduce the behavior of the ODE and hence properties of the mammalian cell cycle. Although the BN is only a simplified approximation of the ODE, the results for individual components in BNE are in agreement. Additionally to the BN fixed points (G0 and 7 periodic attractor), results (Fig. N) of the BNE give insight into the cell status change during G0-phase (PH-276, boolean attractor), G1-phase (PH-260, PH-4, PH-516, PH-518) and start of the S-phase (PH-646).

For Non-boolean values of CycD, CycB's propensity is rather low and can be interpreted as inactive during the accumulation of CycD (CycD $\in (0, 1)$). At the G1 phase Cdh1's propensity is rather high and is as well active in ODE. Similarly Rb's propensity is high at the start of the G1 phase. The propensity of CycE is following the increasing propensity of E2F (Fig. M) as described in the ODE. Additionally, E2F and CycE show a successive activation when mapping to the nearest cell state (Fig. N). However, according to the nearest cell state, their activation is present at the start of the S-phase which actually should be active in the end of G1-phase. In the S/G2/M phases CycA's propensity is increasing and its influence decreases Cdh1 activity which is modelled by the Boolean attractor of length 7. Cdc20's rather low propensity in G1-phase (Fig. N) coincides with the low Cdc20 activity in the ODE model. Its activation is only required to exit the M-phase (Boolean attractor of length 7).

The BN model is a simplification of the ODE cell cycle model. The authors (Faure et al.) also report inconsistency for p27 activation in the model probably due to lack of appropriate interactions in the Boolean Network model which BNE inherits and thus also shows inconsistent behaviour of p27 activity [4].

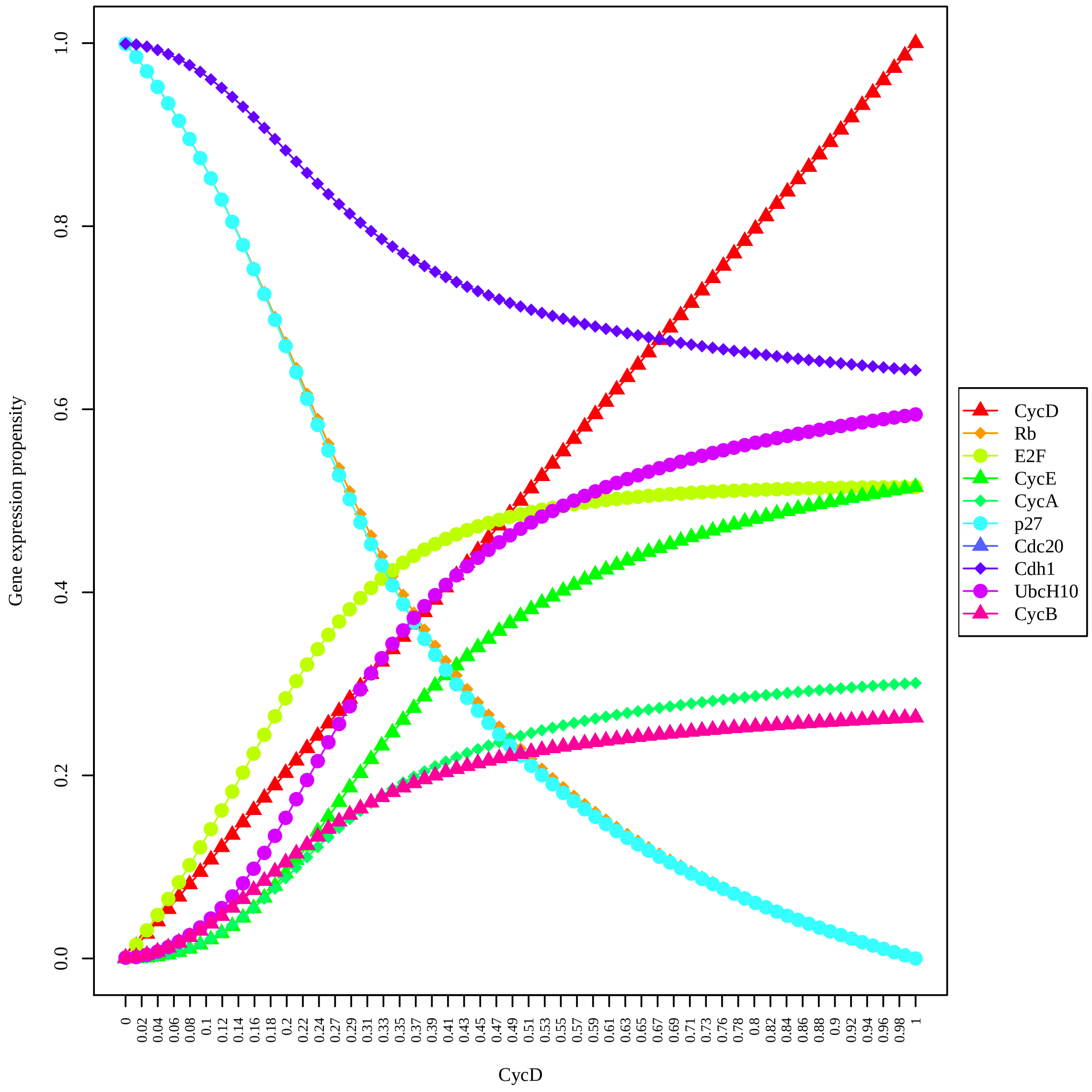

Fig. M. Fixed points for mammalian cell cycle. The figure shows the fixed points in dependence of the CycD start value.

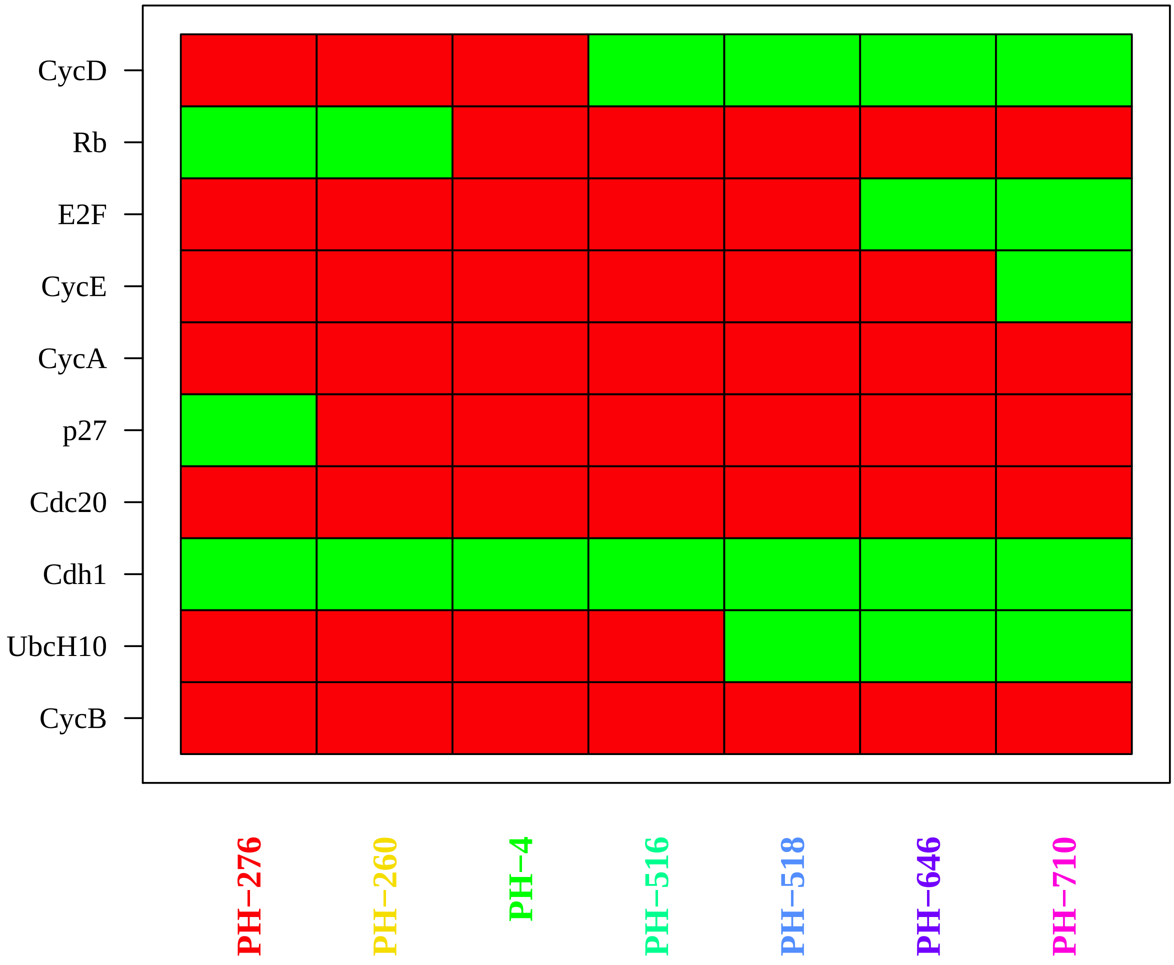

Fig. N. Nearest molecular cell states for the mammalian cell cycle

Table C. Boolean network model of the mammalian cell cycle network: Boolean formulae for the state transitions. The genes of the network are shown in the left column. The subsequent state of each gene is constructed by applying the Boolean function in the right column to the previous state of the genes. Elementary Boolean functions are denoted by $\neg$ = NEGATION, $\vee$ = OR and $\wedge$ = AND. The meaning of the variables can be found in Table 1 in the main text.

**t+1**	**t**
$CycD$	$CycD$
Rb	$(\neg CycD \wedge \neg CycE \wedge \neg CycA \wedge \neg CycB) \vee (p27 \wedge \neg CycD \wedge \neg CycB)$
$E2F$	$(\neg Rb \wedge \neg CycA \wedge \neg CycB) \vee (p27 \wedge \neg Rb \wedge \neg CycB)$
$CycE$	$(E2F \wedge \neg Rb)$
$CycA$	$(E2F \wedge \neg Rb \wedge \neg Cdc20 \wedge \neg(Cdh1 \wedge UbcH10)) \vee (CycA \wedge \neg Rb \wedge \neg Cdc20 \wedge \neg(Cdh1 \wedge UbcH10))$
$p27$	$(\neg CycD \wedge \neg CycE \wedge \neg CycA \wedge \neg CycB) \vee (p27 \wedge \neg(CycE \wedge CycA) \wedge \neg CycB \wedge \neg CycD)$
$Cdc20$	$CycB$
$Cdh1$	$(\neg CycA \wedge \neg CycB) \vee Cdc20 \vee (p27 \wedge \neg CycB)$
$UbcH10$	$\neg Cdh1 \vee (Cdh1 \wedge UbcH10 \wedge (Cdc20 \vee CycA \vee CycB))$
$CycB$	$\neg Cdc20 \wedge \neg Cdh1$

References

1. Herrmann F, Groß A, Zhou D, Kestler HA, Kühl M. A Boolean Model of the Cardiac Gene Regulatory Network Determining First and Second Heart Field Identity. PLoS One. 2012;7:e46798.
2. Müssel C, Hopfensitz M, Kestler HA. BoolNetan R package for generation, reconstruction and analysis of Boolean networks. Bioinformatics. 2010;26(10):1378–1380.
3. Gessert S, Kühl M. Comparative gene expression analysis and fate mapping studies suggest an early segregation of cardiogenic lineages in Xenopus laevis. Developmental Biology. 2009;334:395–408.
4. Fauré A, Naldi A, Chaouiya C, Thieffry D. Dynamical analysis of a generic Boolean model for the control of the mammalian cell cycle. Bioinformatics. 2006;22(14):e124–e131.
5. Novák B, Tyson JJ. A model for restriction point control of the mammalian cell cycle. Journal of Theoretical Biology. 2004;230:563–579.
